# Neutrophil infiltration and microglial shifts in sepsis induced preterm brain injury: pathological insights

**DOI:** 10.1186/s40478-025-02002-2

**Published:** 2025-04-21

**Authors:** Jinjin Zhu, Tiantian He, Ziwei Huang, Wenkai Yu, Jinnan Lu, Shan Zhang, Xiaoli Zhang, Huifang Dong, Yiran Xu, Xiaoyang Wang, Changlian Zhu

**Affiliations:** 1 Henan Key Laboratory of Child Brain Injury and Henan Clinical Research Center for Child Neurological Disorders, Institute of Neuroscience, The Third Affiliated Hospital of Zhengzhou University, Zhengzhou, 450052 China; 2 Centre of Perinatal Medicine and Health, Institute of Clinical Science, University of Gothenburg, Gothenburg, 40530 Sweden; 3 Department of Women’s and Children’s Health, Karolinska Institutet, Stockholm, 17177 Sweden; 4 Center for Brain Repair and Rehabilitation, Institute of Neuroscience and Physiology, University of Gothenburg, Goteborg, 40530 Sweden

**Keywords:** Neonatal sepsis, White matter injury, Neutrophil, Microglial metabolic reprogramming, PANoptosis, Behavioral deficits

## Abstract

Preterm sepsis is a major contributor to brain injury and long-term neurodevelopmental impairments, but its molecular mechanisms remain poorly understood. This study integrated clinical and experimental approaches to investigate the pathological changes linking systemic inflammation to brain injury in preterm infants. Transcriptomic analysis of septic preterm infants’ peripheral blood revealed upregulated immune, metabolic, and inflammatory pathways, suggesting a link between systemic and brain inflammation. Using P2 mice, we established a preterm white matter injury model through multiple doses of lipopolysaccharide, observing dose-dependent developmental delays, brain inflammation, and long-term behavioral deficits. Integrative analyses of peripheral blood and brain samples from both mice and preterm infants revealed consistent chemokine alterations and immune cell infiltration across peripheral and central compartments, highlighting the significant involvement of neutrophil extracellular traps in preterm brain injury. Furthermore, microglia exhibited significant transcriptional changes during the acute phase, accompanied by metabolic reprogramming from oxidative phosphorylation to glycolysis, with suggested involvement of Pgk1 and Pgam1. This shift intensified with escalating inflammation, along with PANoptosis-related gene upregulation, ultimately associated with microglial cell death. Collectively, these findings provide pathological insights into the immunometabolic alterations underlying sepsis-induced preterm brain injury and suggest potential targets for future therapeutic interventions to mitigate long-term neurodevelopmental deficits.

**Supplementary Information:**

The online version contains supplementary material available at 10.1186/s40478-025-02002-2.

## Background

Despite advances in neonatal intensive care, the incidence of neurodevelopmental impairment in preterm infants remains high, particularly in those affected by perinatal infection and sepsis [25, 37, 46]. Preterm sepsis is a major contributor to morbidity and mortality in extremely low birth weight infants, largely due to their underdeveloped immune systems [54]. Although these infants primarily depend on innate immunity, their innate immune functions are also compromised, exhibiting reduced neutrophil migration, impaired phagocytosis, and inadequate secretion of chemokines and cytokines [41, 58]. This immune dysfunction increases the risk of systemic inflammation, a key factor implicated in white matter injury (WMI) [17], the most prevalent form of brain damage in preterm infants. The late gestation and early postnatal periods are critical for oligodendrocyte progenitor cell (OPC) differentiation and myelination [13]. Disruption of this process—commonly associated with preterm birth—significantly impairs white matter development and increases the risk of injury [3]. Currently, treatment strategies for WMI remain limited, and the molecular pathways linking systemic inflammation to WMI are not yet fully understood.

Microglial has been recognized as a central factor in the pathogenesis of preterm WMI [2]. Under physiological conditions, microglia primarily rely on oxidative phosphorylation (OXPHOS) for immune surveillance and homeostasis [18]. However, upon inflammatory stimulation, microglia shift their metabolism from OXPHOS to glycolysis [61]. This shift not only provides rapid energy but may also exacerbate brain inflammation. Both in vitro and in vivo studies demonstrate that, under pathological conditions, microglia reprogram their metabolism to enhance pro-inflammatory gene expression or modulate phagocytic function, thus regulating brain inflammation [3, 21]. In preterm neonates, this metabolic shift may be heightened due to immature mitochondrial function and diminished antioxidant capacity [49]. However, whether a similar metabolic reprogramming occurs in inflammation-induced preterm brain injury remains unclear.

Unlike in adult brain injury models, depleting microglia in immature brains has shown limited neuroprotective effects and may even exacerbate injury [4]. This may be due to microglia’s critical roles in early neurodevelopment, including synaptic pruning, neural circuit modulation, OPC differentiation, and white matter development [6, 62]. Severe inflammation can also activate multiple cell death pathways in macrophages, including PANoptosis, a process that integrates pyroptosis, apoptosis, and necroptosis [38]. Although studies on PANoptosis have shed light on the molecular interactions among these cell death pathways, its role in preterm brain injury remains underexplored [63]. Given microglia’s dual function in immune regulation and neurodevelopment, microglial death during early brain maturation may exacerbate inflammation and tissue damage, leading to more severe long-term consequences [52].

Increasing evidence from adult studies indicates that systemic inflammation induced microglial reactivity and promotes substantial neutrophil infiltration into injured brain regions, thereby exacerbating brain damage [12]. In hypoxia–ischemia (HI) brain injury, neutrophils rapidly infiltrate the immature central nervous system, releasing reactive oxygen species (ROS), neutrophil extracellular traps (NETs), and matrix metalloproteinase-9 (MMP-9) [67]. These factors disrupt the blood–brain barrier (BBB) and induced microglial reactivity, further aggravating brain inflammation [71]. NETs play a dual role in inflammation, enhancing immune responses under both infectious and sterile conditions [43]. The process termed NETosis depends on NADPH oxidase-derived ROS and requires activation of myeloperoxidase (MPO) and neutrophil elastase [8]. In neonatal sepsis, NETs and related components, including cell-free DNA and citrullinated histone H3 (CitH3), are markedly elevated in peripheral blood and correlate with disease severity [31]. However, whether LPS-induced systemic inflammation likewise triggers extensive neutrophil infiltration and how NETs contribute to preterm brain injury remains insufficiently characterized [15].

In this study, we aimed to elucidate key pathological mechanisms underlying LPS-induced immature brain injury, given the limited effective treatment options for preterm brain inflammation. By integrating clinical data from preterm infants with sepsis—including transcriptomic and Olink analyses—and an LPS-induced neonatal mouse model of white matter injury, we identified critical pathological features such as neutrophil infiltration, NET formation, microglial metabolic reprogramming, and cell death. These findings and their associated transcriptional signatures shed light on the mechanisms responsible for sepsis-associated encephalopathy (SAE) and provide a foundation for developing targeted interventions to mitigate long-term neurodevelopmental deficits in preterm infants.

## Materials and methods

### Clinical sample acquisition and processing

This study received approval from the Ethics Committee of The Third Affiliated Hospital of Zhengzhou University (2018-04), and written informed consent was obtained from the parents of all participating infants. Preterm infants in the sepsis group were diagnosed by neonatologists, while a control group of 29 non-infected preterm infants, hospitalized during the same period, was selected and matched for gestational age. Blood samples were collected from the sepsis group within 12 h of diagnosis and from the control group at a comparable postnatal age. Basic information regarding the included samples is summarized in Table S1, with pathogen distribution information provided in the supplementary materials.

### Inclusion and exclusion criteria for sepsis in preterm infants

This study included preterm infants (gestational age < 37 weeks) who met the established diagnostic criteria for sepsis [14]. Sepsis was categorized as either clinically diagnosed or laboratory-confirmed. The clinical diagnostic criteria were as follows: Abnormal clinical manifestations with at least one of the following conditions: (1), at least two positive results from non-specific blood tests, including white blood cell count (WBC) < 5 × 10⁹/L or > 20 × 10⁹/L, C-reactive protein (CRP) ≥ 10 mg/L, serum procalcitonin > 0.5 mg/L, or platelet count < 100 × 10⁹/L; (2), cerebrospinal fluid analysis indicating purulent meningitis; (3), detection of pathogenic bacterial DNA in blood through molecular methods. The laboratory diagnostic criteria required the presence of clinical manifestations along with positive blood or cerebrospinal fluid cultures. All included infants had abnormal clinical signs; some met clinical criteria, and others were confirmed by positive cultures.

Exclusion criteria included preterm infants with severe complications or genetic abnormalities, such as chromosomal or genetic defects, cyanotic congenital heart disease, gastrointestinal abnormalities, or other congenital malformations requiring surgical correction in the neonatal period.

### Animals

C57BL/6J male and female mice, aged 8–10 weeks, were obtained from Vital River (Beijing, China). The animals were housed in a pathogen-free animal facility at the Third Affiliated Hospital of Zhengzhou University, with free access to food and water, and maintained on a 12-h light‒dark cycle. Both male and female pups were included in this study and were randomly assigned to groups stratified by sex. All procedures followed the institutional animal care standards set by the Ethics Committee of Zhengzhou University [Ethics number 2022-(yu)121]. The sample size was determined based on our previous experiment [68], and power analysis was performed using GPower software (v. 3.1.9). Littermates were randomly assigned to different experimental groups. All age- and weight-matched animals with both sexes were included. The complete experimental procedure is illustrated in the Results section.

### LPS administration

To induce sepsis in neonatal mice, lipopolysaccharide (LPS) (*Escherichia coli* 0111: B4, L2630, Source #0000097043, Sigma) was used. Only pups weighing 1.5–2.0 g at postnatal day 2 (P2) were included. LPS was subcutaneously injected between the shoulders at doses of 2.5, 5, and 10 mg/kg (in a volume of 10 µl/g) in sterile saline, while saline- injected mice served as controls (10 µl/g). Mouse survival, body weight, and appearance were monitored at various time points, as depicted in the Results section.

### Flow cytometry and cell sorting

Mouse pups underwent transcardial perfusion with cold normal saline (NS) at 24 h, 72 h, or 5 days post-LPS induction. Brain tissues were enzymatically dissociated using a Neural Tissue Dissociation Kit (130092628, Miltenyi) and mechanically dissociated with a gentleMACS™ Dissociator (Miltenyi, Biotec). The cells were filtered through a 70-µm strainer and resuspended in 30% Percoll. Mononuclear cells were isolated by centrifugation at 300 × g for 30 min at room temperature. The cells were blocked with anti-mouse CD16/CD32 and stained with FVS-BV510 for viability testing. Mononuclear cells were stained following standard protocols, and the antibody information is listed in Table S2. The data were acquired using a FACSCanto II (BD Biosciences) and analyzed with FlowJo v10.0 software.

Brain tissues (including the meninges, but excluding the cerebellum and hydrangea) from each group were dissociated into single cells as described. CD45^+^ cells were isolated using the EasyStep Mouse CD45-Positive Selection Kit (STEMCELL Technologies). The sorting purity is shown in Fig. S2A-C. CD45^+^ cell pellets were stored at − 80 °C for subsequent RNA extraction.

### RNA sequencing (RNA-seq)

In this study, 16 RNA-seq replicates from clinical samples were divided equally into two groups. Whole blood was used for transcriptome sequencing, with basic information regarding the included samples summarized in Table S1. For the animal experiments, 32 RNA-seq replicates were used, with 8 replicates for each group, balanced for sex. Each replicate corresponded to a single animal. The CD45^+^ cell precipitate isolated from mouse brains was homogenized in RLT lysis buffer for RNA extraction. RNA library construction and sequencing were conducted by Hangzhou Lianchuan Biological Information Technology Co., Ltd. (Hangzhou, China). The transcriptome sequencing data are available in the BioSample database (BioProject ID: PRJNA1061839). Details on sample preparation, sequencing depth, and quality control are provided in the supplementary materials.

### Luminex assay

Mouse pups underwent intracardial perfusion with cold saline. Their brains (including the meninges, excluding the cerebellum and hippocampus) were harvested at 24 h and 72 h, post-LPS injection and stored at − 80 °C. After thawing, the brains were homogenized using a Tissuelyser grinder (Jingxin, China) and mixed with 250 µl of cell lysis buffer containing a protease inhibitor cocktail (Roche, USA). Protein quantification was performed using bicinchoninic acid reagent (P0012S, Beyotime, China) according to the manufacturer’s instructions. The absorbance was measured at 560 nm using a Spark 10 M spectrophotometer (Tecan, Switzerland). Whole blood from mice was collected by cardiac puncture, and plasma was obtained after centrifugation at 500 × g for 10 min and frozen until analysis. All samples were assayed with the Mouse Chemokines Discovery Luminex Performance Assay Kit (RD Systems) using a Luminex200 system (Nasdaq, USA). Chemokine levels in brain tissue were quantified as picograms per milligram of total protein (pg/mg).

### Proteomic analysis

We reanalyzed our previous proteomic data [14] with a focus on assess neutrophil function in sepsis infants. Proteomic profiling was performed using an Olink inflammation panel (Olink Proteomics AB, Sweden) according to the manufacturer’s instructions, analyzing 29 plasma samples from each patient group. The results are reported as normalized protein expression values on a log2 scale, with higher values indicating increased protein abundance.

### Transmission electron microscopy (TEM)

Mouse pups were perfused with cold saline, followed by immediate immersion in 2.5% glutaraldehyde in phosphate buffer. Coronal Sect. (1 mm thick) were prepared using a McIlwain Tissue Chopper and isolated around the hippocampus region (~ 1 mm³). The experimental procedures were performed according to our previous study [69]. After polymerization, ultrathin sections were cut using an ultramicrotome (Leica EM UC7) and examined under an electron microscope (HT-7800).

### Behavioral analysis

Behavioral responses in mice from P40 to P45 were evaluated using various tests. All experiments were performed at the same time of day (14:00–17:00), and the animals were habituated to the behavioral testing room for one hour prior to testing. The tests were conducted in a quiet, dimly lit environment. The sample size was determined using GPower, aiming for a power of 0.75 and a significance level of 0.05. To minimize animal handling, reduce stress, and avoid task interference, in line with ethical guidelines to prevent overburdening a single animal with multiple tasks, two cohorts were used: one for the O-maze, Y-maze, and rotarod tests, and another for social tasks, novel social partner tests, and passive avoidance tests.

On P40, anxiety in the O-maze test was assessed by measuring the time spent in the open versus enclosed arms [59]. The Y-maze test, conducted on P41, evaluated short-term spatial memory through the percentage of alternations, calculated as (number of alternations/ total number of entries– 2) × 100 [29]. The novel object recognition test on P42, which included habituation, training, and testing phases, quantified explorative behavior using recognition and discrimination indices [69]. Social interactions at P43 were assessed using a three-chamber apparatus, with the sociability index is calculated as (social exploration– empty exploration) / (social exploration + empty exploration) [51]. Motor function was evaluated on P44 using the rotarod test, where the duration of retention on the rod was recorded under an accelerating protocol. All tests were monitored and analyzed using the ANY-maze video tracking system and software. Detailed information on behavioral analysis is provided in the supplementary materials.

### Other analyses

We also performed various other analyses, including (1) immunofluorescence histochemistry staining, (2) western blotting, (3) RT‒qPCR, and (4) RNA‒seq data analysis. The details of these analyses are provided in the supplementary materials.

### Statistical analysis

The results are shown as the mean ± SEM. A heatmap was generated to visualize the saliency of the Luminex assay. IBM SPSS 21.0 (IBM, USA) was used for the statistical analyses. Data normality and variance homogeneity were assessed with Shapiro–Wilk and Levene’s tests. Comparisons between the NS and single LPS groups were conducted using a two-tailed unpaired t test or Mann-Whitney U test. Statistical analysis of data satisfying normality and homogeneity of variances was performed using one-way ANOVA followed by Tukey’s post hoc test. For data not meeting normality assumptions, a Kruskal–Wallis H test followed by Dunn’s post hoc test was employed. For data with unsatisfactory homogeneity of variance, Welch’s ANOVA followed by Games-Howell’s post hoc test was used [55]. Two-way ANOVA with Bonferroni post hoc or Scheirer-Ray-Hare tests with a Kruskal‒Wallis test were performed for comparisons between different time points or sexes within the NS and LPS groups [27]. RNA-seq gene expression data in FPKM were analyzed with DESeq2. P values < 0.05 were considered significant.

## Results

### Transcriptomic profiling reveals key molecular mechanisms of sepsis-related brain injury in preterm infants

**Fig. 1:**
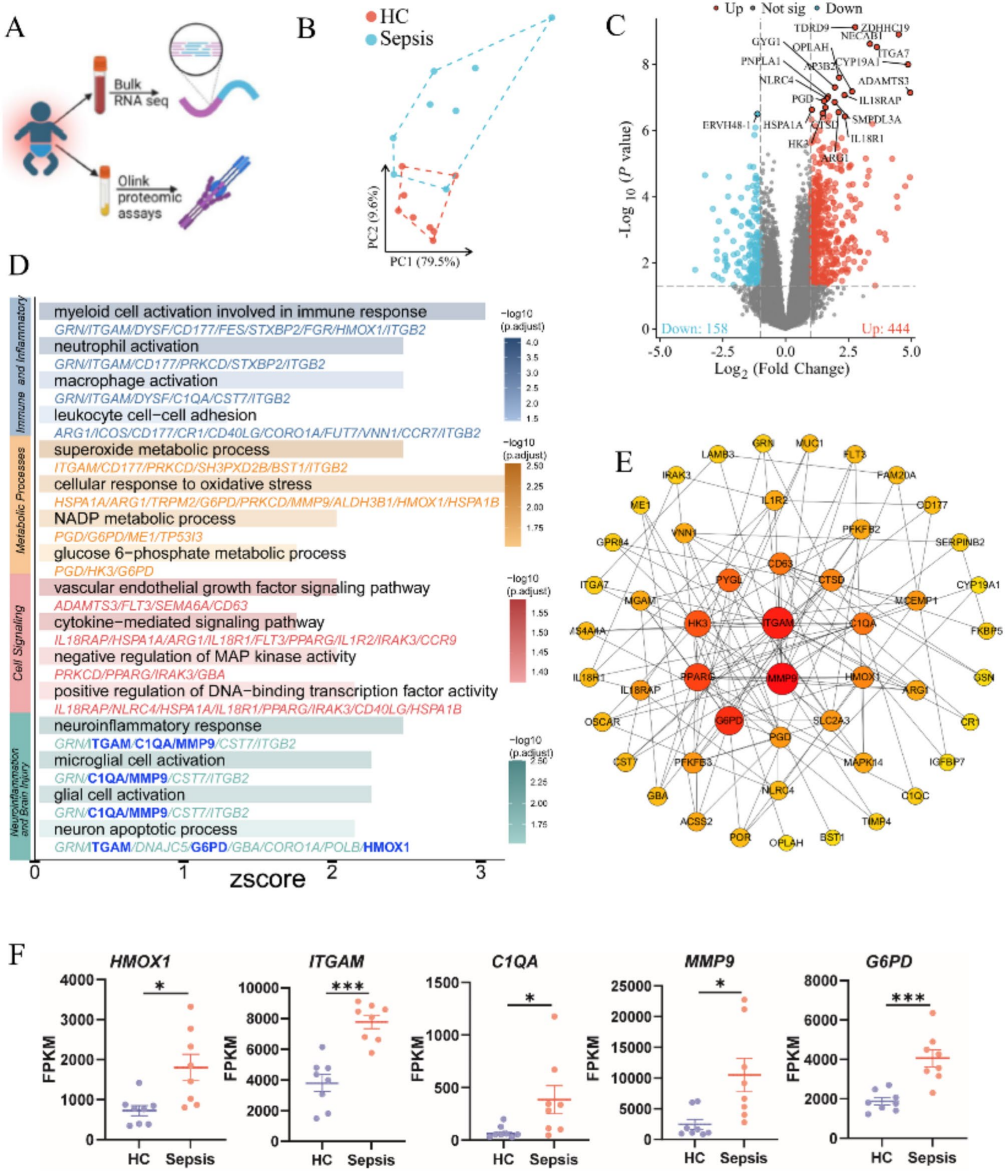
Perinatal LPS Exposure causes developmental delays and white matter injury in neonatal mice. **A**. Experimental timeline overview. **B**. Representative images of neonatal mice at P5 showing physical appearance after treatment with NS, 5 mg/kg, or 10 mg/kg LPS. **C**. Bar graph displaying body weight gain at different time points (P3, P5, and P12) relative to P2. Statistical analysis was performed using One-way ANOVA with Tukey’s post hoc test or Kruskal‒Wallis test with Dunn post hoc test (P3-female: H = 27.3, *P* = 0.000; P3-male: F3,39 = 119.5, *P* = 0.000; P5-female: H = 23.8, *P* = 0.000; P5-male: H = 19.2, *P* = 0.000; P12-female: F3,39 = 13.6, *P* = 0.000; P12-male: F3,42 = 8.5, *P* = 0.000; *n* = 8–15/group, **P < 0.01, ***P < 0.001, NS vs. LPS). **D.** Histogram showing the mortality ratio three days after LPS injection across groups (NS group, *n* = 29; 2.5 mg/kg LPS group, *n* = 59; 5 mg/kg LPS group, *n* = 83; 10 mg/kg LPS group, *n* = 80). **E.** Quantification of MBP-positive areas in the corpus callosum (CC) at P12 (Kruskal‒Wallis test with Dunn post hoc test: H = 25.8, *P* = 0.000, *n* = 9/group, *P* < 0.01, *P < 0.001, NS vs. LPS, #*P* < 0.05, 2.5 mg/kg LPS vs. 10 mg/kg LPS). **F.** Representative MBP-stained images of the CC at P12, showing myelin structure in the NS, 2.5 mg/kg, 5 mg/kg, and 10 mg/kg LPS groups. Scale bars: 1 mm (middle column) and 100 μm (side column). **G**. Quantification of Iba-1-positive cells per unit area (mm²) in the brain 24 h post-LPS treatment (One-way ANOVA with Tukey’s post hoc test: F3,14 = 7.6, *P* = 0.002; *n* = 4–5/group, *P < 0.05, **P < 0.01, NS vs. LPS). **H**. Representative Iba-1 staining (green) at 24 h after LPS treatment for different doses (NS, 5 mg/kg, and 10 mg/kg). Scale bars: 500 μm (left) and 100 μm (right)

We conducted transcriptomic profiling of peripheral blood from septic preterm infants to explore molecular pathways potentially involved in brain injury. Principal component analysis (PCA) revealed distinct gene expression patterns between the sepsis and control groups, with PC1 accounting for 79.5% of the variance (Fig. 1B). Differential expression analysis identified 444 upregulated and 158 downregulated genes in the sepsis group compared to controls (adjusted *P* < 0.05,|log2 fold change| > 1) (Fig. 1 C).

Gene Ontology (GO) enrichment analysis of these differentially expressed genes (DEGs) highlighted immune/inflammatory responses, metabolic processes, cell signaling, and brain inflammation as key pathways linking systemic inflammation to brain injury (Fig. 1 D). Protein-protein interaction (PPI) network analysis using CytoHubba further identified hub genes, including *ITGAM* (associated with immune cell adhesion and migration), *HMOX1* (a regulator of oxidative stress and inflammation), *MMP9* (involved in extracellular matrix degradation and blood-brain barrier (BBB) disruption), *C1QA* (a critical component of the complement system), and *G6PD* (essential for maintaining cellular redox balance and protecting against oxidative damage), which are involved in brain inflammation, BBB disruption, and oxidative stress (Fig. 1E, F), suggesting that systemic inflammation may contribute to brain injury in these infants.

To explore these findings in more depth, we employed a established LPS-induced systemic inflammation model in neonatal mice [45, 68], with further characterization,

to investigate the molecular mechanisms underlying brain injury in a controlled setting.

### LPS-induced white matter injury and developmental delays in neonatal mice

**Fig. 2:**
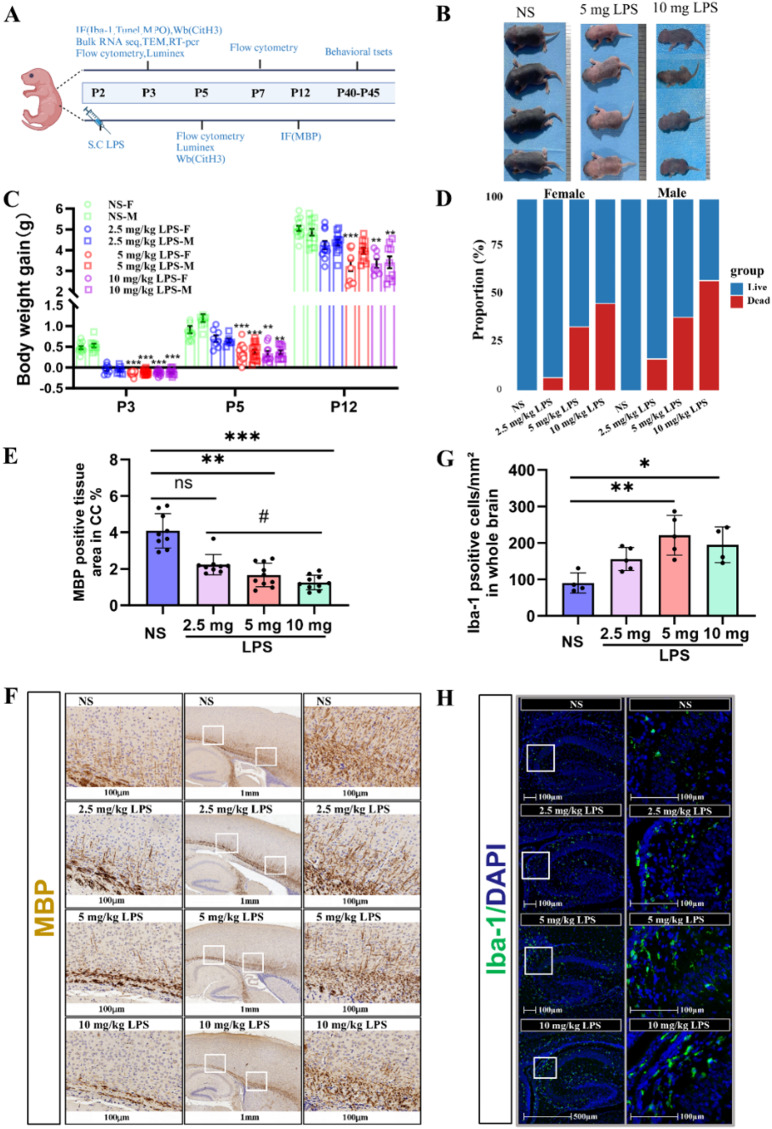
Transcriptomic analysis uncovers key inflammatory pathways and hub genes in sepsis. **(A)** Overview of experimental workflow for the transcriptome analysis and Oink proteomic assay in sepsis preterm infants. **(B)** PCA plot displaying the transcriptomic separation between the sepsis and healthy control (HC) groups (*n* = 8/group). **(C)** Volcano plot illustrating differential gene expression between the sepsis and HC groups, with log2 fold change (FC) on the x-axis and − log10 (q-value) on the y-axis. The top 20 most significant differentially expressed genes (DEGs) are labeled. **(D)** Bar plot showing the GO term enrichment analysis of DEGs, with Z-scores indicating the direction and magnitude of pathway regulation. The color gradient reflects the significance (-log10(p. adjust)). **(E)** Circular layout depicting the top 50 hub genes identified by the STRING/Cityscape/cytoHubba interface. The size and color of each node (larger size and deep red) correspond to the gene rank based on the degree algorithm in the cytoHubba plug-in. **(F)** Comparative expression levels of selected hub genes (HMOX1, ITGAM, C1QA, MMP9, G6PD) between the HC and sepsis groups (*n* = 8/group), analyzed using two-tailed unpaired t-tests or Mann–Whitney U tests (*HMOX1*: t = 3.04, *p* = 0.014; *ITGAM*: t = 5.63, *p* = 0.000; *C1QA*: U = 53, *p* = 0.027; *MMP9*: t = 2.83, *p* = 0.021; *G6PD*: t = 4.59, *p* = 0.000). *P < 0.05, **P < 0.01, ***P < 0.001

To model neonatal sepsis and its impact on brain development, we administered varying doses of LPS to P2 mice. Mice treated with higher doses of LPS (5 mg/kg and 10 mg/kg) exhibited significant developmental delays and body weight reduction by P5 compared to controls, whereas the 2.5 mg/kg group showed no notable effects (Fig. 2 B, C). Increased mortality rates were observed in a dose-dependent manner, with the highest dose (10 mg/kg) group showing the greatest mortality, particularly within the first 24 h post-LPS administration (Fig. 2 D).

Histological analysis revealed dose-dependent white matter injury, as indicated by reduced myelin basic protein (MBP) expression in the corpus callosum at P12 (Fig. 2 E, F). Brain inflammation was also evident, with increased Iba-1 positive microglia in the white matter regions of LPS-treated mice at P3 (Fig. 2 G, H). These findings suggest that perinatal LPS exposure induces dose-dependent white matter injury, brain inflammation, and developmental delays.

### Long-term neurobehavioral effects of perinatal LPS exposure

Preterm infants with sepsis may exhibit neurobehavioral impairments, though the underlying link remains unclear. It is also unknown whether LPS-induced white matter injury in neonatal mice leads to long-term brain damage and specific neurological deficits. To investigate this, we used a 5 mg/kg LPS dose, which caused notable brain injury without high mortality. Both sexes were included to assess potential sex differences, as males appear more susceptible to neurodevelopmental disorders [39].

**Fig. 3:**
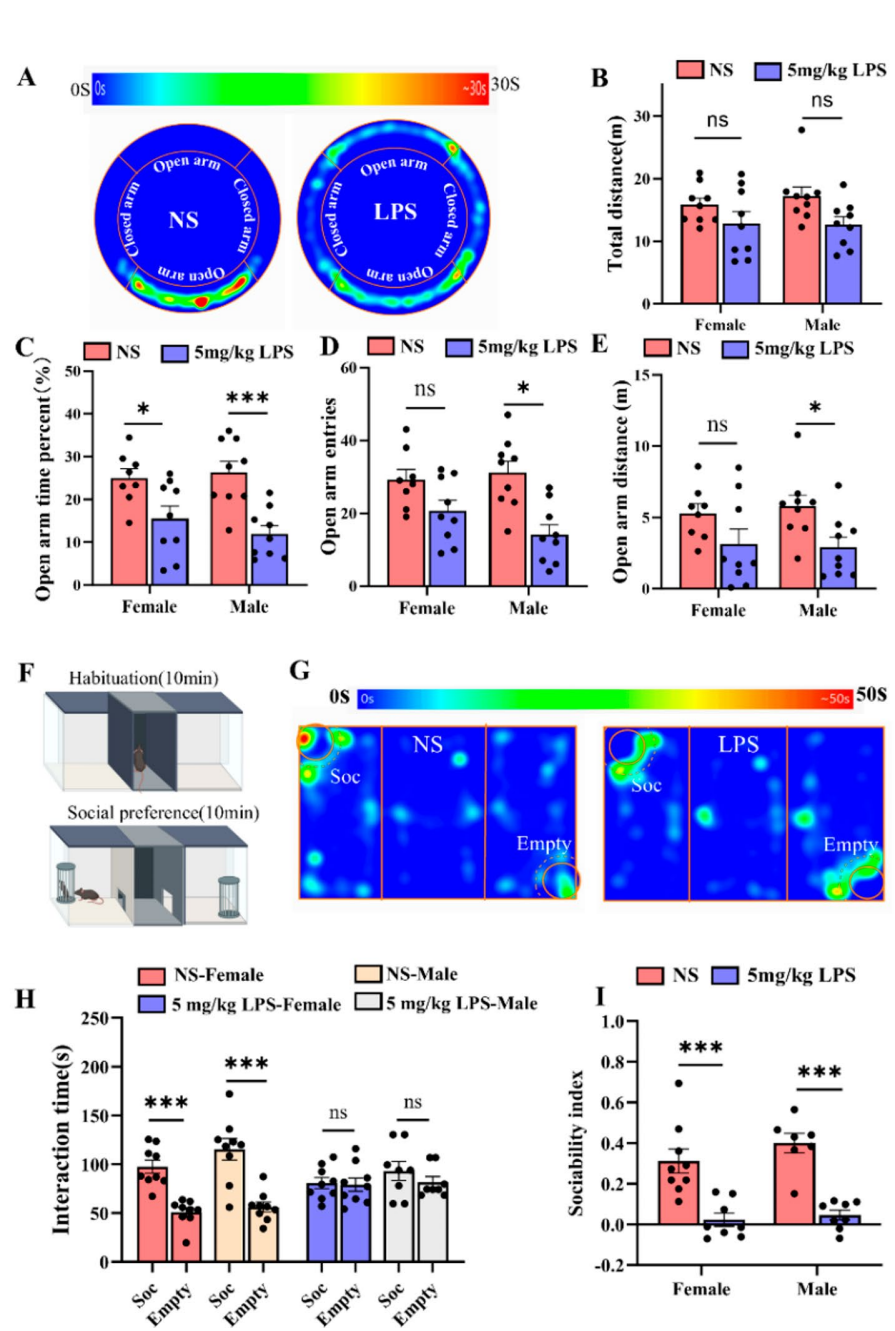
Perinatal LPS exposure induces long-term anxiety-like behavior and social deficits. **A.** Heatmaps showing the movement distribution of NS- and LPS-treated mice during the O-maze test. **B-E.** Bar graphs illustrating the total distance traveled, number of open arm entries, percentage of time spent in the open arms, and distance traveled in the open arms during the O-maze test. Statistical analysis was performed using two-way ANOVA with Bonferroni post hoc correction. Results are as follows: total distance: no LPS effect: F_1,35_ = 2.6, *P* = 0.11; no sex effect: F_1,35_ = 0.4, *P* = 0.49; no interaction: F_1,35_ = 0.5, *P* = 0.47. Time percent in open arms: LPS effect: F_1,35_ = 23.3, *P* = 0.000; no sex effect: F_1,35_ = 1.1, *P* = 0.73; no interaction: F_1,35_ = 1.3, *P* = 0.25. Open arm entries: LPS effect: F_1,35_ = 18.0, *P* = 0.000; no sex effect: F_1,35_ = 0.423, *P* = 0.53; no interaction: F_1,35_ = 1.3, *P* = 0.25. Distance in open arms: LPS effect: F_1,35_ = 8.7, *P* = 0.006; no sex effect: F_1,35_ = 0.08, *P* = 0.77; no interaction: F_1,35_ = 0.3, *P* = 0.56 (*n* = 9/group, *P < 0.05, ***P < 0.001). **F.** Two-phase social preference (S-E) protocol: a 10-min habituation phase followed by a 10-min test with a social stimulus (Soc: mouse under a cup) and a nonsocial stimulus (Empty: empty cup). **G.** Heatmaps displaying the time distribution in the social preference test for NS- and LPS-treated mice. **H**. Bar graphs showing the time spent interacting with the social stimulus (Soc) or the empty cup (Empty) for NS- and LPS-treated mice. Statistical analysis with Student’s unpaired t test: NS-female: t = 5.7, *P* = 0.000; NS-male: t = 7.1, *P* = 0.000; LPS-female: t = 0.3, *P* = 0.75; LPS-male: t = 0.8, *P* = 0.42 (*n* = 7–9/group). **I**. Social preference indices for NS- and LPS-treated mice, analyzed by Two-way ANOVA with Bonferroni post hoc correction: Social index: LPS effect: F_1,31_ = 42.6, *P* = 0.000; no sex effect: F_1,31_ = 0.2, *P* = 0.64; no interaction: F_1,31_ = 1.31, *P* = 0.26 (*n* = 7–9/group). *P < 0.05, ***P < 0.001

We conducted a series of neurobehavioral tests on mice at P40. Mice treated with 5 mg/kg LPS exhibited increased anxiety-like behavior, as evidenced by reduced time spent in the open arms of the O-maze. Interestingly, males showed reductions across all measures, females exhibited a significant decrease only in open arm time. (Fig. 3A-E) [59]. Social interaction deficits were also observed, with LPS-treated mice displaying reduced preference for social stimuli (Fig. 3F-I).

**Fig. 4:**
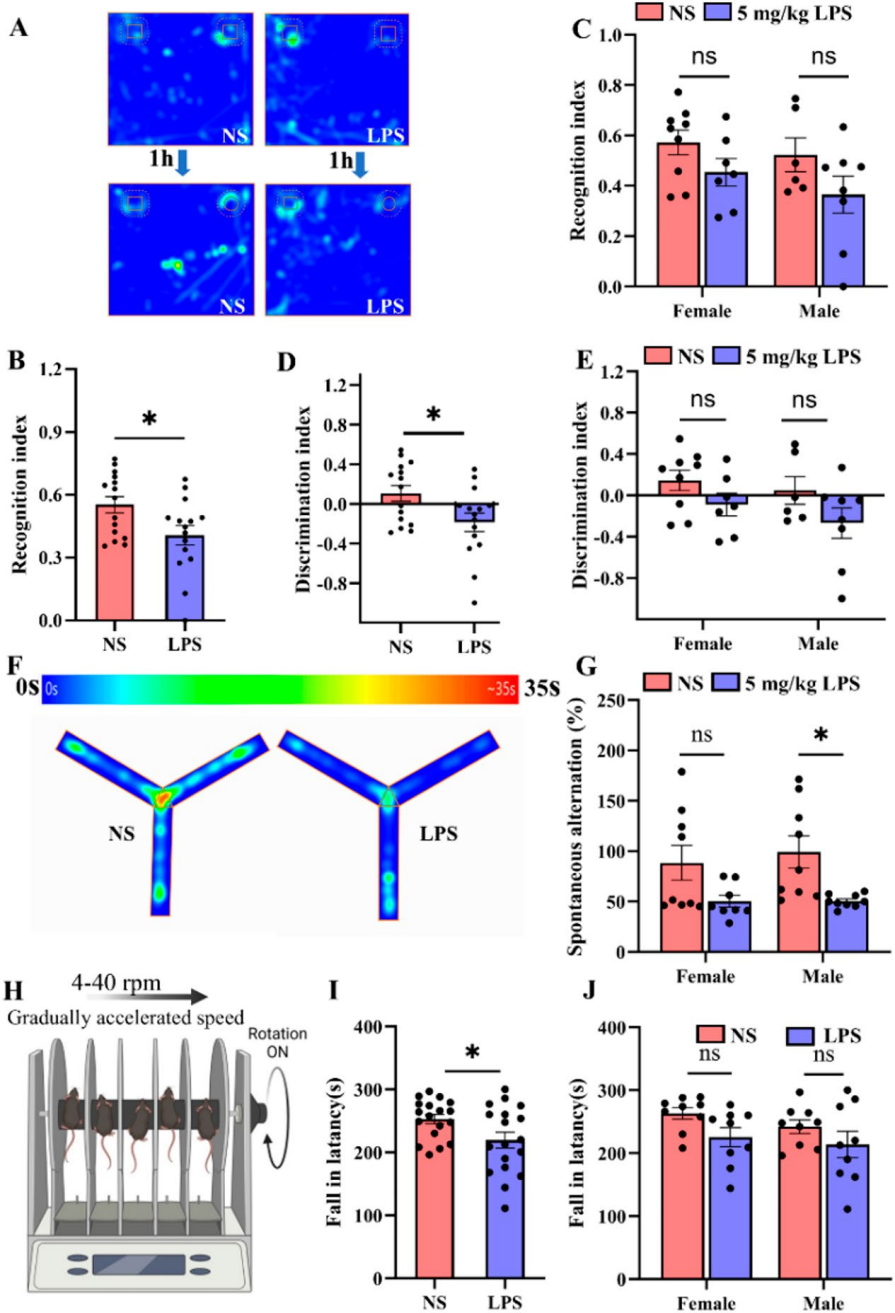
Perinatal LPS exposure moderately impairs motor and cognitive functions. **A.** Representative heatmaps illustrating the head movements of mice during the novel object recognition test, comparing exploration of a familiar object versus a novel object. **B-E.** Bar graphs showing the recognition index (novel object time/total exploration time) and the discrimination index [(novel object time − familiar object time)/total exploration time]. Two-way ANOVA with Bonferroni post hoc correction (**C**, **E**) revealed a significant effect of LPS on the recognition index (F_1,29_ = 4.9, *P* = 0.035), but no effect of sex (F_1,29_ = 1.231, *P* = 0.27) and no interaction (F_1,29_ = 0.103, *P* = 0.75). Similarly, the discrimination index showed a significant LPS effect (F_1,29_ = 4.933, *P* = 0.035), with no effect of sex (F_1,29_ = 1.232, *P* = 0.27) and no interaction (F_1,29_ = 0.103, *P* = 0.75) (*n* = 6–9/group). For individual comparisons, Student’s unpaired t test (**B**, **D**) indicated significant differences in both the recognition index (t = 2.4, *P* = 0.02) and discrimination index (t = 2.4, *P* = 0.02). **F**. Representative heatmaps showing tracking patterns over 5-minute in the Y-maze. **G.** Bar graphs showing the percentage of spontaneous alternations calculated in the Y-maze test [(number of alternations/total number of entries − 2) × 100]. The Scheirer-Ray-Hare test with Kruskal‒Wallis test for pairwise comparisons showed a significant LPS effect (H = 9.0, *P* = 0.002), but no effect of sex (H = 1.7, *P* = 0.18) or interaction (H = 0.2, *P* = 0.62) (*n* = 9/group). **H**. Rotarod test setup. **I-J.** Bar graphs showing the latency to fall in both NS and LPS groups. Student’s unpaired t test for (I) and two-way ANOVA with Bonferroni post hoc correction for (J revealed an LPS effect (F_1,35_ = 5.0, *P* = 0.03), with no effect of sex (F_1,31_ = 1.2, *P* = 0.27) and no interaction (F_1,31_ = 0.1, *P* = 0.75; t = 2.2, *P* = 0.03) (*n* = 9/group). * *P* < 0.05

In the novel object recognition test (Fig. 4A-E), LPS-treated mice showed impaired learning and memory, with lower recognition and discrimination indices compared to controls without sex difference (Fig. 4B-E). In the Y-maze test (Fig. 4F), spontaneous alternation, which reflects spatial memory, was reduced in LPS-treated mice, with males being more affected (Fig. 4G). Motor function, assessed using the rotarod test at P44, was also impaired in LPS-treated mice, suggesting long-term motor impairment (Fig. 4H-J). These results indicate that perinatal LPS exposure leads to lasting neurobehavioral impairments, including anxiety, social deficits, cognitive and motor dysfunction.

### Parallel immune cell infiltration and chemokine dynamics in LPS-treated mice and preterm infants

**Fig. 5:**
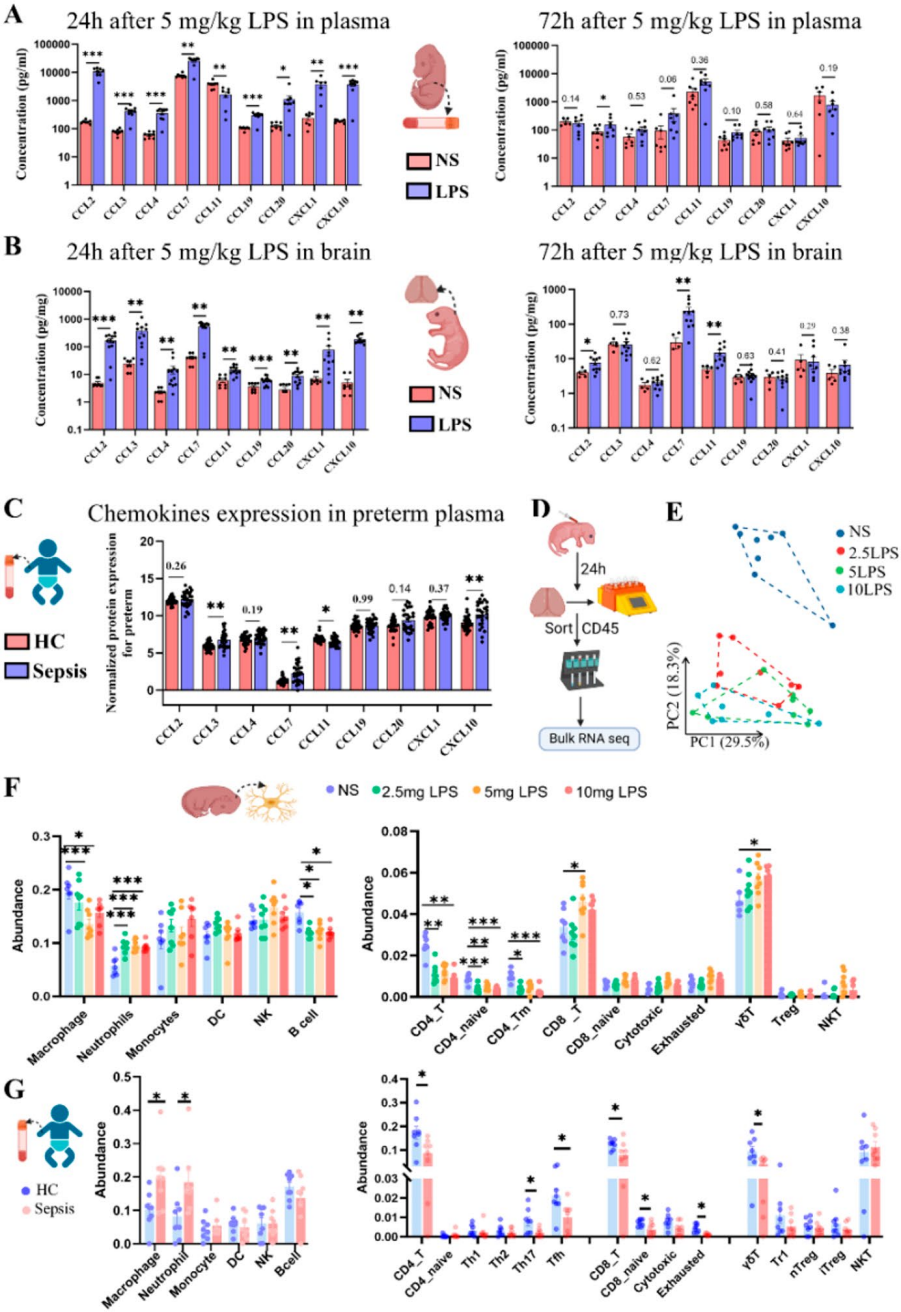
Chemokine expression and immune cell infiltration in LPS-treated neonatal mice and preterm infants with sepsis. **A-B**. Chemokine levels in neonatal mouse plasma (**A**) and brain tissue (**B**) measured at 24 h and 72 h after 5 mg/kg LPS treatment (*n* = 5–10/group). Statistical analyses were performed using t-tests, ANOVA, or Kruskal-Wallis tests with appropriate post hoc corrections. Full results are presented in Table S4-S5. **C**. Normalized chemokine protein expression in plasma from preterm infant (HC vs. Sepsis), measured using the Olink proteomics platform (*n* = 27–29/group). **D.** Workflow for transcriptome analysis of immune cells using magnetic bead sorting. **E.** PCA plot showing the transcriptomic separation among the groups (*n* = 7–8/group). **F-G**. Immune cell infiltration analysis based on bulk RNA-seq data, using the ImmuCellAI database for preterm infants (**F**) and LPS-treated neonatal mice (**G**) (*n* = 7–8/group). *P < 0.05, **P < 0.01, ***P < 0.001

To investigate the link between the dynamics of chemokine changes and immune cell infiltration into the brain, we conducted a joint analysis integrating data from septic preterm infants and LPS-treated neonatal mice. In both septic infants and 5 mg/kg LPS-treated mice, peripheral blood showed an acute inflammatory chemokine response (Fig. 5A, C). CCL3, CCL7, and CXCL10 were significantly elevated in both species, whereas CCL11 (eotaxin) decreased markedly, indicating a conserved early response pattern. Notably, the mouse peripheral response was broader, with additional increases in CCL2, CCL4, CCL19, CCL20, and CXCL1, which remained unchanged in infants. This indicates that while the core chemokine response is similar, its magnitude and range are more pronounced in the murine model.

At 24 h post-LPS, chemokine changes in mouse blood and brain were largely parallel. Eight chemokines (including CCL2, CCL3, CCL4, CCL7, CCL19, CCL20, CXCL1, and CXCL10) were elevated in both plasma and brain tissue, reflecting a synchronized peripheral and central response (Fig. 5B). CCL11 was the exception, decreasing in peripheral blood but increasing in the brain. Similarly, sepsis preterm infants showed a CCL11 decrease in blood, suggesting shared systemic regulation. The compartment-specific upregulation of CCL11 in the brain highlights a distinct brain inflammatory regulation within CNS.

By 72 h, most peripheral chemokines had returned to baseline, except for persistently elevated CCL3. In contrast, brain levels of CCL2, CCL7, and CCL11 remained high. This temporal divergence suggests that central inflammation persists beyond peripheral resolution, indicating prolonged brain inflammation in the neonates.

At 24 h post-LPS, transcriptomic analysis of CD45⁺ cells in the neonatal mouse brain (Fig. 5D, E) revealed substantial immune-related gene expression changes. Leveraging RNA-seq and ImmuCellAI, we observed increased neutrophils in both mouse brain and septic infants’ peripheral blood (Fig. 5F–G), reflecting a heightened innate immune response. However, T cell subsets diverged: in mice, CD8⁺T cells (5 mg/kg) and γδ T cells (10 mg/kg) increased, while CD4⁺T cells declined; in infants, both CD4⁺ and CD8⁺T cells decreased in the blood, suggesting potential tissue infiltration.

In summary, in both septic preterm infants and LPS-treated neonatal mice, peripheral chemokines exhibit similar patterns of change, whereas brain inflammation persists longer in mice than the peripheral response, indicating prolonged brain inflammation.

### Transcriptomic and network analyses reveal shared and dose-specific inflammatory signatures in preterm sepsis and LPS-treated neonatal mice

**Fig. 6:**
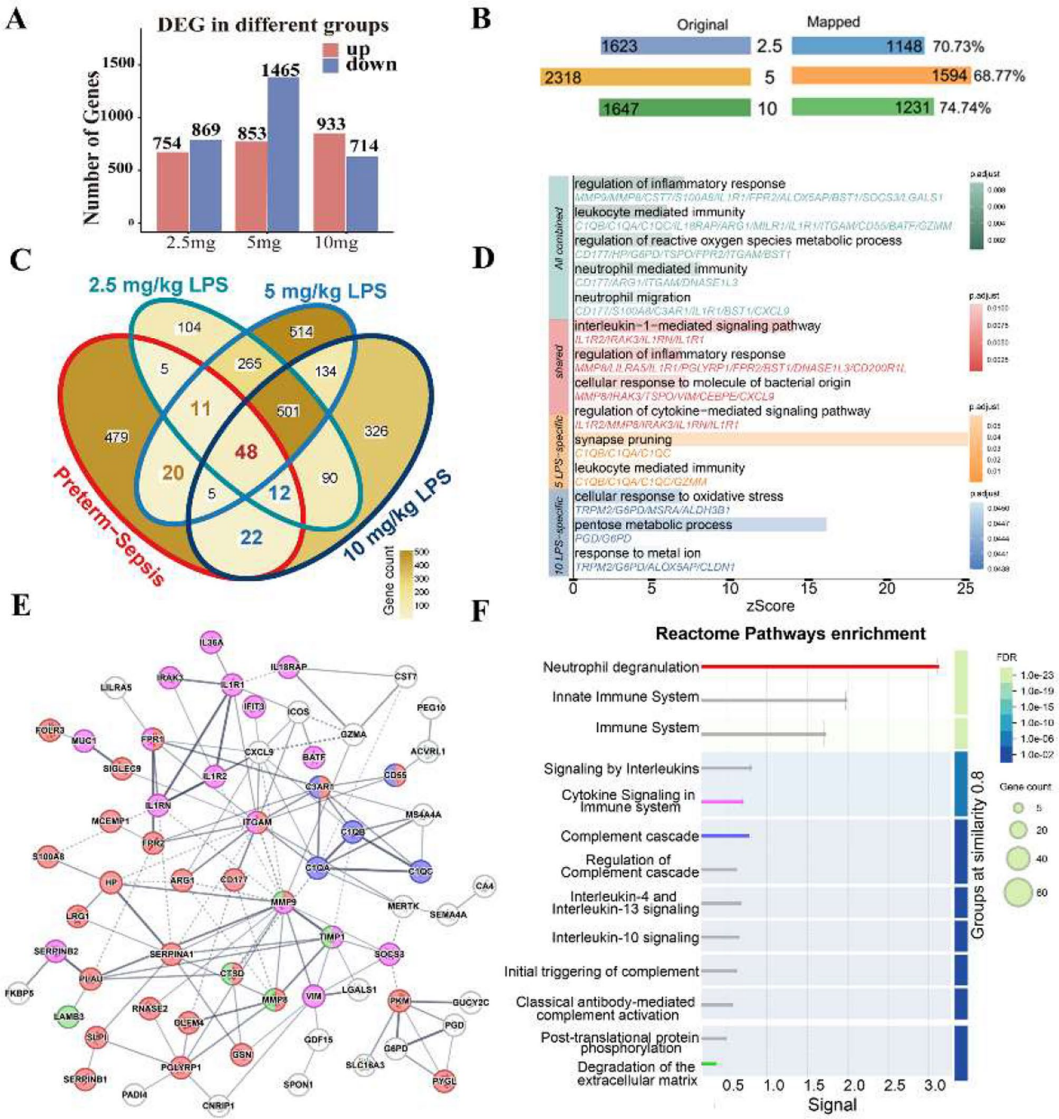
Differentially expressed genes (DEGs) and network analysis in preterm sepsis and LPS-treated neonatal mice. **A**. Bar chart displaying the number of DEGs in each LPS treatment groups. **(B)** Gene mapping diagram showing the conversion of mouse DEGs to human genes across different LPS treatments. **(C)** Venn diagram showing DEGs overlap between the preterm sepsis group and various LPS treatment groups. **(D)** Bar plot of GO term enrichment analysis for each DEGs set. All combined group (green): Intersection of 123 DEGs between the preterm sepsis and all LPS treatment groups. Shared group (red): 48 DEGs common to both the preterm sepsis and all three LPS treatment groups. 5 mg/kg LPS-specific group (yellow): 20 uniquely overlapping genes. 10 mg/kg LPS-specific group (blue): 22 genes uniquely overlapping genes. **(E)** PPI network derived from the “All combined” DEGs using the STRING database. Circles indicate proteins, and lines represent predicted interactions; the line thickness reflecting the confidence level of interaction. **(F)** Reactome pathway enrichment analysis corresponding to E. Each pathway is color-matched to its respective protein nodes in E

To investigate molecular overlaps between preterm sepsis and LPS-treated mice, we first identified DEGs across various LPS treatment groups (adjusted *P* < 0.05,|log2 fold change| > 1) (Fig. 6A). We then mapped the mouse genes to their human orthologs (68–74% mapping) for cross-species comparisons (Fig. 6B).

The Venn diagram (Fig. 6C) illustrates the DEG overlap between preterm sepsis and each LPS treatment group and GO analysis (Fig. 6D) reveals distinct functional profiles. The “All combined” group is enriched in pathways related to inflammatory responses and neutrophil migration, highlighting the overall molecular reaction to systemic inflammation. In contrast, the “Shared” group predominantly comprises genes involved in IL-1–mediated signaling and cytokine production, reflecting a core inflammatory profile common to both preterm sepsis and all LPS doses. Moreover, the gene set unique to the 5 mg/kg LPS group is enriched in synapse pruning pathways, suggesting a potential impact on neurodevelopment, while the 10 mg/kg LPS-specific group is enriched for genes associated with oxidative stress response and metal ion homeostasis, indicating a dose-dependent effect on inflammatory and metabolic regulation.

To clarify the mechanistic roles of these “All combined” genes, we constructed a PPI network and performed Reactome pathway enrichment analysis (Fig. 6E-F). Most of these genes (red) were linked to neutrophil degranulation pathways, whereas others were related to cytokine signaling and complement activation. Notably, several key hub genes involved in neutrophil function were identified: *ITGAM* and *C3AR1* mediate neutrophil adhesion and chemotaxis [7], *MMP9* facilitates infiltration via extracellular matrix degradation, and *SERPINA1* modulates protease activity to protect tissues during inflammation [40]. Overall, our integrated analysis of peripheral blood from septic preterm infants and LPS-exposed neonatal mouse brains suggests that peripheral inflammation may drive brain injury by promoting neutrophil infiltration and triggering metabolic disruption.

### LPS-induced neutrophil infiltration and NET formation in the neonatal mouse brain

**Fig. 7:**
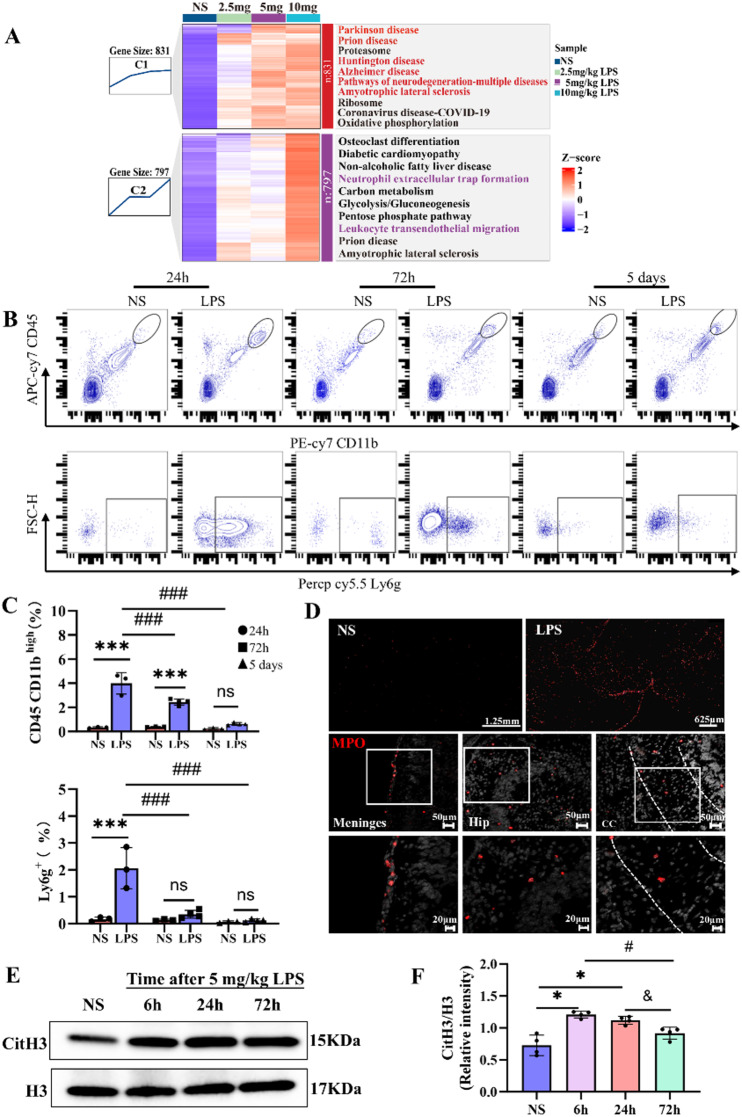
Neutrophil infiltration and NETs formation induced by LPS in the neonatal mouse brain. **A**. Mfuzz clustering heatmap showing dynamic expression patterns across the four groups. The top 10 KEGG pathways are displayed with their z-scores (blue: negative, red: positive). Cluster 1 and Cluster 2 highlight the differential effects of various LPS doses. Genes name are listed in Table S6**B**. Flow cytometry gating strategy used to analyze immune cells in the peripheral blood. **C**. Bar graphs showing immune cell (CD45^high^/CD11b^+^) and neutrophil (CD45^high^/CD11b^+^/Ly6g^+^) expression in the neonatal mouse brain at 24 h, 72 h, and 5 days after 5 mg/kg LPS treatment. Analysis was performed using the Scheirer–Ray–Hare test with Kruskal–Wallis corrections. For Ly6g: LPS effect: H = 6.3, *P* = 0.011; time effect: H = 9.0, *P* = 0.010; no interaction: H = 0.5, *P* = 0.76. For CD45CD11b: LPS effect: H = 14.6, *P* = 0.000; no time effect: H = 3.1, *P* = 0.20; no interaction: H = 0.6, *P* = 0.73 (*n* = 3–4/group, ***P *<* 0.001, NS vs. LPS, ^###^*P* < 0.001 24 h vs. 72 h/5 days for LPS). **D**. Representative MPO (red) staining in the NS and 5 mg/kg LPS groups at 24 h (scale bars: 50 μm upper, 20 μm lower). **E-F.** Western blot quantification of CitH3 in neonatal mouse brain lysates at 6 h, 24 h, and 72 h after 5 mg/kg LPS treatment. H3 was used as the loading control. Welch one-way ANOVA test with Games–Howell post hoc test: F_3,11_ = 14.9, *P* = 0.002 (*n* = 4/group, *P *<* 0.05, NS vs. LPS, ^#^P *<* 0.05, 6 h vs. 72 h, ^&^P *<* 0.05, 24 h vs. 72 h)

To investigate the relationship between LPS dose-dependent gene expression patterns and their associated functions, we categorized all genes into seven clusters based on their expression profiles using Mfuzz, followed by Kyoto Encyclopedia of Genes and Genomes (KEGG) pathway enrichment analysis for each cluster (Fig. S1A-B). Notably, the characteristics of Cluster 1 and Cluster 2 showed a high dependence on LPS dose (Fig. 7A). Cluster 1 was enriched in processes related to neuronal damage, while Cluster 2 was associated with leukocyte migration and NET formation—web-like structures released by neutrophils to capture pathogens—and crucial for bacterial defense [32]. These findings suggest that neutrophils may play a key role in LPS-induced brain injury.

Indeed, flow cytometry analysis of neonatal mouse brains 24 h post-LPS revealed increased infiltration of myeloid cells (CD45⁺/CD11b⁺) and neutrophils (Ly6G⁺) (Fig. 7B-C). Immunofluorescence confirmed prominent neutrophil infiltration (MPO-positive) not only in the meningeal vessels but also in the brain parenchyma (hippocampus and corpus callosum) (Fig. 7D). Western blot analysis of Cit-H3, a marker of NET formation [28], revealed elevated levels in the 5 mg/kg LPS group at 6 and 24 h, with a return to baseline at 72 h (Fig. 7E-F). These results indicate significant neutrophil infiltration and NET formation in the neonatal brain following systemic inflammation induced by LPS.

### Microglial metabolic reprogramming: Glycolysis as a key driver of inflammation

**Fig. 8:**
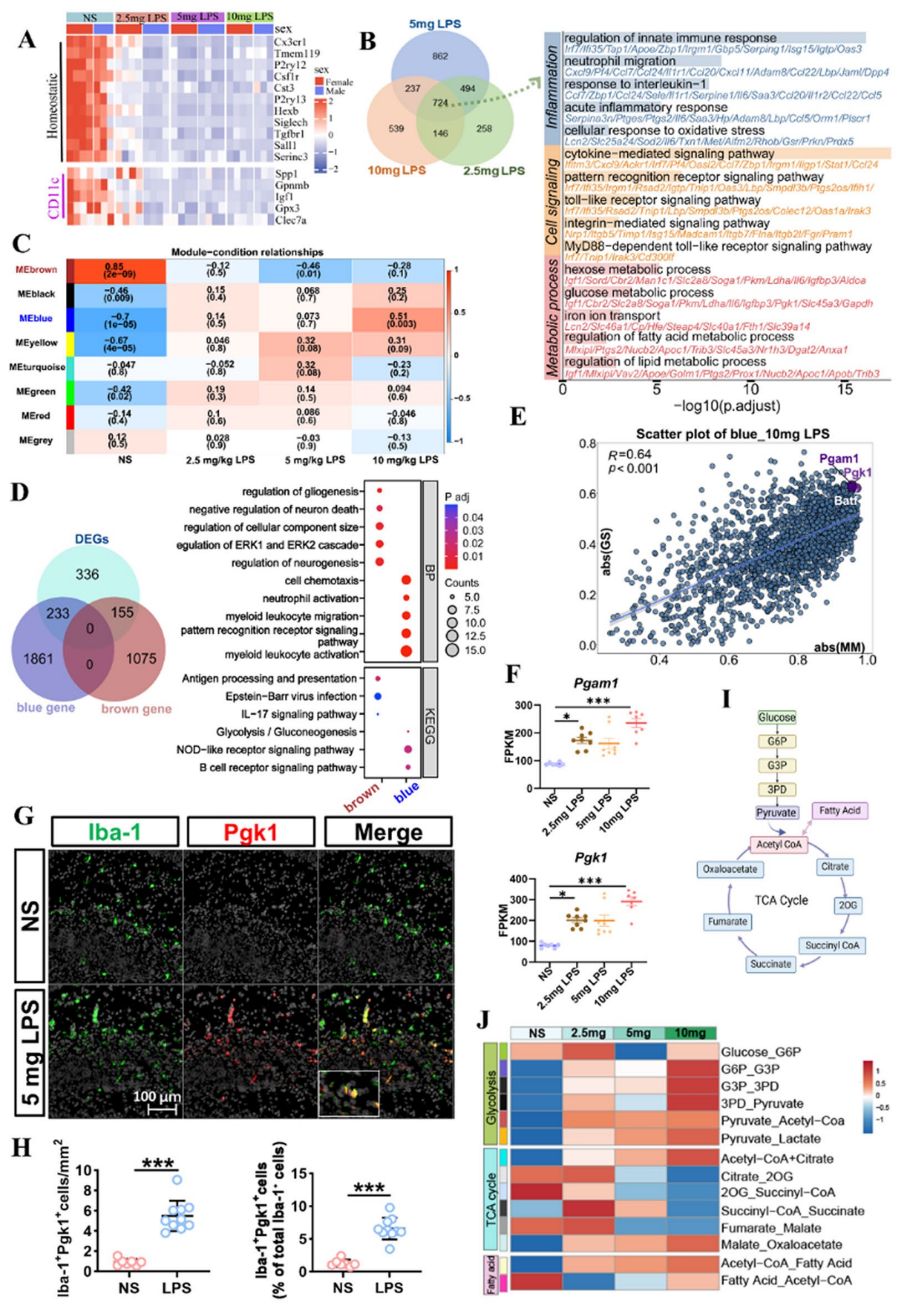
LPS-Induced metabolic and homeostatic dysregulation in neonatal microglia. **(A)** Heatmap illustrating changes in microglia-related gene expression following LPS treatment. **(B)** Bar graph showing the number of DEGs in each LPS dosage group compared to NS, accompanied by a Venn diagram indicating the overlap of DEGs across the groups. A total of 724 common DEGs were used for GO term enrichment analysis, with the bar plot displaying the results and x-axis representing -log10(p.adjust). **(C)** WGCNA module-condition relationship heatmap, highlighting the brown and blue modules as being strongly correlated with LPS treatment. **(D)** Venn diagram showing the overlap of the 724 common DEGs with the brown and blue modules, followed by a dot plot of enriched biological processes and pathways. **(E)** Scatter plot illustrating gene significance (GS) and module membership (MM) in the blue module for the 10 mg/kg LPS group, highlighting key metabolic genes. **(F)** Bar graphs showing Pgk1 and Pgam1 expression across treatment groups. Kruskal–Wallis test followed by Dunn’s post hoc test: Pgk1: H = 19.978, *p* = 0.0002; Pgam1: H = 20.65, *p* = 0.0010 (*n* = 7–8/group, *P < 0.05, ***P < 0.001, NS vs. LPS). **(G)** Representative immunofluorescence images of Iba-1 (green) and Pgk1 (red) in in P3 mouse brains treated with NS or LPS (5 mg/kg). Scale bar = 100 μm. **(H)** Quantification of Iba-1⁺Pgk1⁺ cell density (cells/mm², left) and the percentage of Pgk1⁺Iba-1⁺ cells among total Iba-1⁺ cells (right). Welch’s t-test (left, t = 9.29, *p* < 0.001) and unpaired t-test (right, t = 8.08, *p* < 0.001). (*n* = 7–10/group, ***p < 0.001). **(I)** A simplified metabolic pathway schematic is displayed on the right (created using BioRender).**J.** Heatmap showing the predicted metabolic flux profile for energy-related modules, generated using scFEA. Each row represents the flux of a specific metabolic module across different cell groups (x-axis), with the color scale bar indicating the magnitude and direction of flux

Next, we investigated the changes in microglia following LPS exposure, as flow cytometry and sequencing confirmed that around 95% of the CD45⁺cells post-LPS exposure were microglia (Fig. S2A-C). Heatmap analysis of DEGs revealed significant downregulation of homeostatic microglial genes, indicating a disruption of microglial homeostasis (Fig. 8A). Notably, genes crucial for white matter development, such as *Igf1*,* Gpnmb*, and *Clec7a* [5, 34], were markedly downregulated in the 5 mg/kg and the 10 mg/kg LPS groups (Fig. S2D-E).

GO enrichment analysis of DEGs highlighted inflammatory and metabolic as core pathways in microglia, particularly lipid and glucose metabolism (Fig. 8B). Using Weighted Gene Co-expression Network Analysis (WGCNA), we identified two key gene modules: the blue module, positively correlated with LPS exposure, and the brown module, negatively correlated, linked to immune regulation and neurodevelopment (Fig. 8C, D). Module analysis identified Pgk1 (phosphoglycerate kinase 1) and Pgam1 (phosphoglycerate mutase 1), key glycolytic enzymes [74], as the most significantly altered genes, indicating enhanced glycolytic metabolism (Fig. 8E, F). To validate this finding, we used Iba-1 to label microglia and found a significant increase in Pgk1-positive microglia at 24 h post 5 mg/kg LPS stimulation (Fig. 8G, H).

Single-cell Flux Estimation Analysis (scFEA) confirmed a shift toward glycolysis in microglia (Fig. 8I-J) [70], characterized by elevated levels of glycolytic intermediates, including glyceraldehyde 3-phosphate (G3P) and pyruvate, alongside reduced tricarboxylic acid (TCA) cycle reactivity (Fig. 8J). Altogether, such metabolic shift may suggest that microglia increasingly rely on glycolysis to fuel the energy demands of inflammation, potentially contributing to their functional dysregulation and exacerbate white matter injury.

### PANoptosis reactivity and microglial cell death in high-dose LPS exposure

**Fig. 9:**
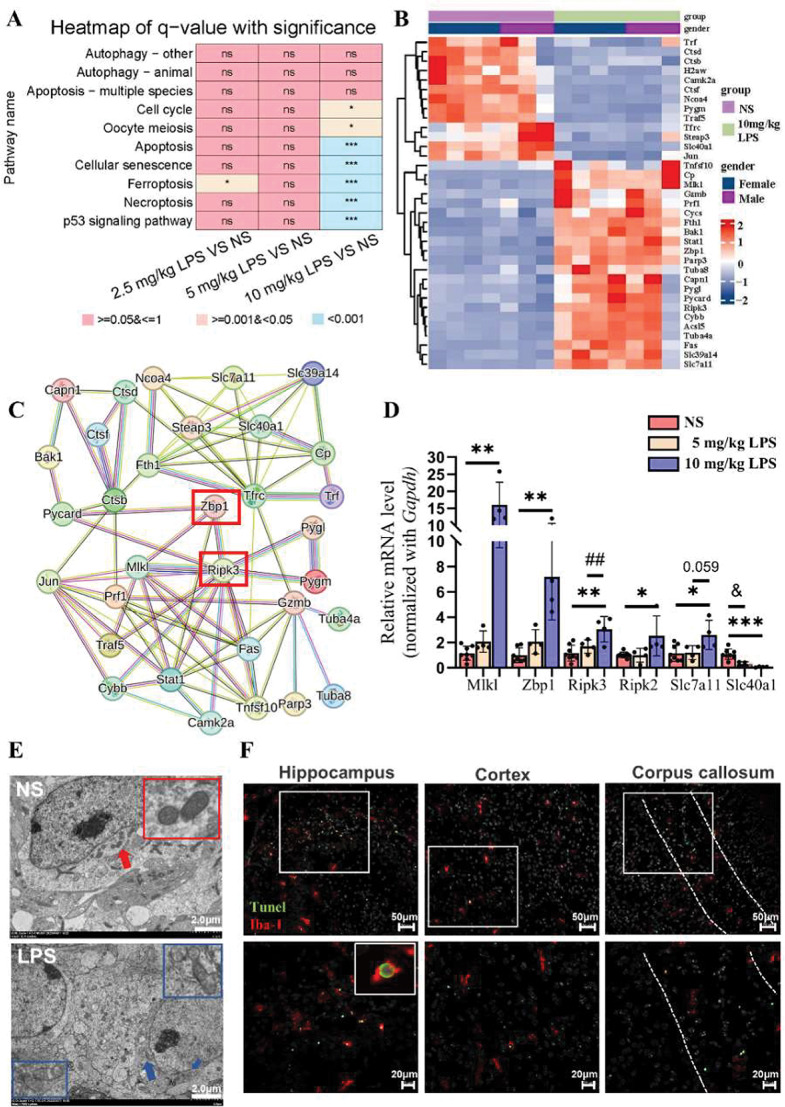
LPS induces microglial programmed cell death in neonatal mice at 24 h. **A**. Heatmap displaying q-values from the KEGG cellular process enrichment analysis with significant pathways indicated (*P < 0.05, ***P < 0.001, NS vs. LPS). **B**. Heatmap showing differential gene expression related to the programmed cell death signaling pathway in neonatal mice exposed to 10 mg/kg LPS. **C**. PPI network of of cell death regulatiory proteins, extracted from the KEGG and STRING databases. Line thickness represents interaction confidence levels (thicker lines indicate higher confidence). **D**. RT-PCR validation of key programmed cell death genes in mouse brain tissue 24 h after LPS injection. Statistical tests used include one-way ANOVA with Tukey’s post hoc test or Kruskal–Wallis test with post hoc Dunn test (*Mlkl*: H = 10.4, *P* = 0.002; *Zbp1*: H = 5.4, *P* = 0.02; *Ripk3*: F_2,13_ = 9.5, *P* = 0.002; *Ripk2*: H = 8.6, *P* = 0.013; *Slc7a11*: F_2,13_ = 5.0, *P* = 0.02; *Slc40a1*: F_2,13_ = 17.0, *P* = 0.000. *n* = 4–8/group). Significant results: ^&^*P* < 0.05, NS vs. 5 mg/kg LPS, * *P* < 0.05, ** *P* < 0.01, *** *P* < 0.001, NS vs. 10 mg/kg LPS, ^##^*P* < 0.01, 5 mg/kg LPS vs. 10 mg/kg LPS. **E**. Representative electron microscope images of mitochondria in control and LPS-treated mice at 24 h after 10 mg/kg LPS treatment. Red and blue arrows indicate normal and damaged mitochondria, respectively (Scale bar = 200 nm). *n* = 3–4/group. **F**. Representative TUNEL (green) and Iba-1 (red) double-staining images of the 10 mg/kg LPS group at 24 h. Scale bars, 50 μm (upper panel) and 20 μm (lower panel)

KEGG pathway analysis in the 10 mg/kg LPS group revealed enrichment in cell death-related pathways (Fig. 9A), with significant upregulation of genes involved in apoptosis, necroptosis, and ferroptosis (Fig. 9B), collectively known as PANoptosis. Key regulators of PANoptosis, such as *Zbp1* (Z-DNA binding protein 1) and *Ripk3*, were significantly upregulated, indicating a convergence of cell death mechanisms in response to severe inflammation (Fig. 9C, D).

TEM images of LPS-treated brain tissues revealed characteristic features of ferroptosis in microglia, including outer mitochondrial membrane rupture, increased electron density, and structural disorganization (Fig. 9E) [33]. TUNEL staining further confirmed microglial cell death in key brain regions, including the hippocampus, cortex, and corpus callosum (Fig. 9F). These findings highlight the role of PANoptosis in microglial cell death and suggest that metabolic reprogramming may increase the susceptibility of microglia to inflammatory cell death.

## Discussion

**Fig. 10:**
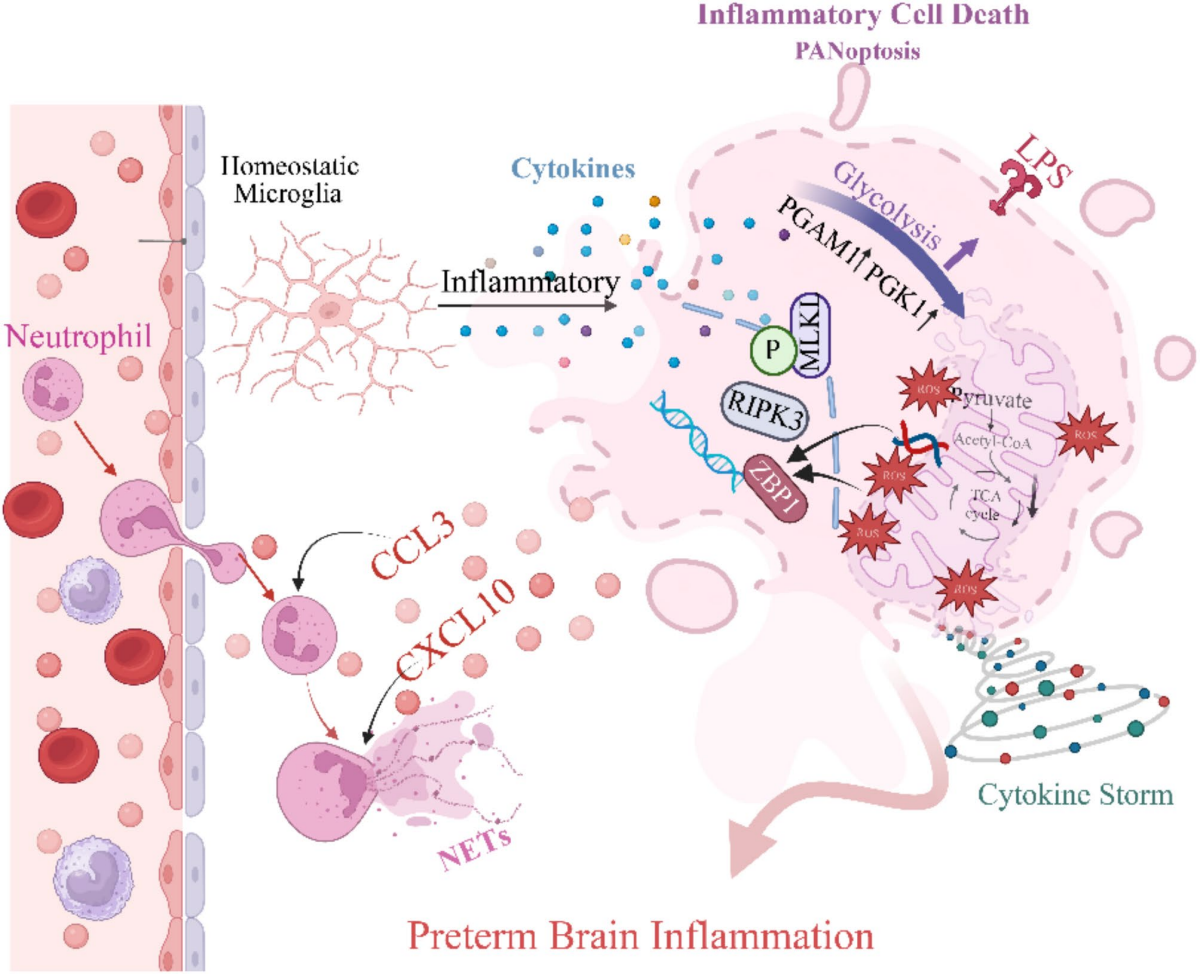
A schematic overview of possible mechanisms underlying systemic inflammation-induced WMI in preterm infants. Systemic inflammation in preterm infants promotes microglial metabolic reprogramming, shifting from oxidative phosphorylation to glycolysis via Pgam1 and Pgk1, and is associated with local inflammation and ROS release. Under severe inflammation conditions, mitochondrial damage worsens, releasing mtDNA and ROS, potentially activating the ZBP1-RIPK3-MLKL pathway and leading to microglial cell death and an amplified inflammatory response. Meanwhile, elevated chemokines drive neutrophil infiltration and NET formation, exacerbating BBB disruption and sustained brain inflammation, ultimately resulting in white matter injury (created using BioRender)

This study comprehensively analyzed transcriptomic data from septic preterm infants’ peripheral blood and from an LPS-induced neonatal mouse sepsis model. We aimed to investigate potential correlation among sepsis-induced systemic inflammation, WMI, and long-term neurobehavioral deficits. Our findings suggested that sepsis concurrently activated peripheral and central immune responses. During the initial phase of inflammation, we observed extensive neutrophil infiltration in the meningeal vessels and brain parenchyma, accompanied by the formation of NETs. Meanwhile, microglia underwent significant metabolic reprogramming, as evidenced by the upregulation of PGK1 and PGAM1. These results provide novel insights into the mechanisms underlying SAE. Nonetheless, further studies are required to confirm causality and clarify the specific roles of these processes, thereby laying a foundation for new interventions aimed improving neurodevelopmental outcomes in preterm infants (Fig. 10).

Early systemic inflammation disrupts white matter development, leading to long-term neurobehavioral deficits and distinct damage patterns in rodent models. In our study, P2 mice exposed to LPS exhibited white matter damage, along with anxiety-like behaviors and social deficits. Similarly, Favrais et al. observed that neonatal mice exposed to inflammatory factors between P1 - P5 suffered white matter damage and cognitive impairments, whereas exposed to IL-1β between P6 - P10 did not produce comparable effects [16]. These diverse findings demonstrate the importance of inflammation timing in neurodevelopmental outcomes [60]. Clinically, newborns with intrauterine infections face a higher risk of autism spectrum disorder (ASD) [1], and neuropathological analyses of ASD patients reveal brain inflammation and microglial reactivity in multiple brain regions [56]. We propose that early systemic inflammation triggers local inflammatory responses and disrupt synaptic homeostasis and neural connectivity, ultimately leading to neurodevelopmental dysfunction [22]. Consequently, strategies aimed at reducing brain inflammation and promoting OPC differentiation are emerging as promising approaches for preventing neurodevelopmental disorders in preterm infants.

Our study identified sepsis-induced neutrophil infiltration and chemokine upregulation as potential links between systemic infection and brain injury. In both neonatal mouse sepsis model and septic preterm infants, CCL3, CCL7, and CXCL10 were similarly up-regulated in blood and brain, promoting neutrophil migration across the BBB and exacerbating brain inflammation. Specifically, CCL3 recruits neutrophils into the brain via CCR1 [50], whereas CCL7 attracts monocytes and mediates neutrophil migration via CCR2-dependent pathway [23]. Toll-like receptor activation further increases CCR2 expression on neutrophils, increasing sensitivity to CCL7 and facilitating brain infiltration [53]. This systemic to CNS inflammatory pathway offers potential biomarkers for the early detection of sustained brain inflammation. For example, CXCL10 levels in blood or cerebrospinal fluid may help identify high-risk neonates and guide timing of clinical interventions [11, 44].

Additionally, our results show that LPS-induced neutrophil infiltration extends beyond the meningeal vessels into the brain parenchyma, contributing to tissue damage. Activated neutrophils release proteases and NETs through NADPH oxidase-mediated oxidative stress, thereby compromising BBB integrity [36]. Among these proteases, MMP-9 is particularly important, as its upregulation closely correlates with BBB disruption [57]. Beyond direct BBB damage, neutrophils also modulate brain inflammation by regulating microglial reactivity. In neonatal HI models, neutrophil depletion has been shown to reduce excessive microglial reactivity and neuronal degeneration [42]. Notably, several experiments highlight the importance of depletion timing: two studies reported neuroprotective effects when neutrophils were depleted 4–12 h post-HI [42, 47], whereas another study showed benefit only when depletion occurred 4 h before HI, with no significant effect when initiated post-insult [67]. However, the role of neutrophils in immature brains subjected solely to peripheral inflammation remains unclear. Given the immaturity of the neonatal immune system and the association of neutropenia with adverse outcomes in preterm infants [48], complete neutrophil depletion may carry significant risks. Therefore, targeting neutrophil infiltration or overactivation presents a safer and more feasible therapeutic approach. Indeed, CXCR2 inhibitors (e.g., AZD-5069) effectively block neutrophil infiltration in inflammatory disorders [64], suggesting their potential application in neonatal brain injury.

Using scFEA, we observed that LPS-induced systemic inflammation shifted microglial metabolism from OXPHOS to glycolysis. Consistent with previous studies, LPS stimulation in vitro increases glycolysis in BV2 cells and primary microglia, promoting M1 polarization and enhancing IL-1β and TNF-α release [21, 61]. These inflammatory cytokines impair OPC function: IL-1β inhibits OPC differentiation [35], while TNF-α induces oligodendrocyte apoptosis [10]. Furthermore, we found elevated Pgk1 and Pgam1 expression in LPS-treated neonatal mice. As a rate-limiting enzyme in glycolysis, the upregulation of Pgk1 significantly enhances ATP production and exacerbates M1 polarization [9]. In contrast, Pgam1 maintains glycolytic homeostasis and mitigates excessive inflammatory responses resulting from metabolic imbalances [24]. These findings suggest that Pgk1 and Pgam1 regulate microglial metabolism in neonatal brain injury, warranting further investigation.

KEGG pathway enrichment analysis showed that PANoptosis-related genes were upregulated only in the 10 mg/kg LPS group. TEM and histological examinations confirmed microglial cell death indeed occurred under these conditions. Previous studies indicate that PANoptosis is activated in various infectious and inflammatory diseases, such as sepsis, viral infections, and autoimmune disorders, and may contribute to retinal and ischemic brain injuries [30, 65, 66]. However, its role in neonatal brain injury remains unclear. Studies suggest that in HI and WMI models, microglia trigger severe brain inflammation via caspase-1-dependent pyroptosis [20, 72], releasing IL-1β and IL-18, which impair OPC differentiation and maturation [26]. In neonatal SAE models, cortical neurons undergo PANoptosis via the TLR9 the MAPK pathway, thereby worsening brain damage [73]. Our findings suggest that Zbp1 and Ripk3 may play pivotal roles in microglial PANoptosis [30], though further study is needed to elucidate how these factors participate in PANoptosis signaling cascades and brain inflammation.

Despite thoroughly characterizing the key aspects of sepsis-related white matter injury and neurobehavioral abnormalities, our study has several limitations. First, LPS-induced mouse model replicates the main inflammatory features of preterm sepsis, it does not fully capture its clinical complexity. Second, while existing research supports a critical role for glycolysis in microglial function, current detection methods limit direct in vivo assessment of microglial metabolism [19]. Transcriptomic analysis reflects gene expression levels without directly measuring metabolic fluxes or activities. Future studies should combine in vivo and in vitro experiments to systematically investigate the roles of PGK1 and PGAM1 in regulating microglial energy metabolism and their contributions to white matter injury. Finally, although neutrophils likely contribute to BBB disruption and white matter injury, their depletion was not tested due to their essential role in neonatal immunity and the potential safety risks. Our findings rely primarily on observational data.

## Conclusion

In summary, our study highlights microglial metabolic reprogramming and neutrophil infiltration as key roles in the pathogenesis of sepsis-induced WMI in preterm infants. A deeper understanding of these processes may facilitate the identification of therapeutic targets to alleviate long-term neurodevelopmental impairments in this vulnerable population. Future research should focus on translating these findings into clinical strategies for early intervention and prevention of sepsis-related neonatal brain injury.

## Electronic supplementary material

Below is the link to the electronic supplementary material.

Supplementary Material 1

Supplementary Material 2

Supplementary Material 3

## Data Availability

The transcriptome sequencing data are available in the BioSample database (BioProject ID: PRJNA1061839).

## Abbreviations

BBB: blood-brain barrier
CC: corpus callosum
Cit: H3-citrullinated histone H3
CRP: c-reactive protein
DEGs: differentially expressed genes
FC: fold change
G3P: glyceraldehyde 3-phosphate
GO: gene ontology
GS: gene significance
HC: healthy control
HI: hypoxia-ischemia
KEGG: kyoto encyclopedia of genes and genomes
LPS: lipopolysaccharide
MBP: myelin basic protein
MM: module membership
MMP-9: matrix metalloproteinase-9
MPO: myeloperoxidase
mtDNA: mitochondrial DNA
NETs: neutrophil extracellular traps
NS: normal saline
OPC: oligodendrocyte progenitor cell
OXPHOS: oxidative phosphorylation
PCA: principal component analysis
Pgam1: phosphoglycerate mutase 1
Pgk1: phosphoglycerate kinase 1
PPI: protein-protein interaction
RNA: seq-RNA sequencing
ROS: reactive oxygen species
RT-PCR: reverse transcription polymerase chain reaction
SAE: sepsis-associated encephalopathy
scFEA: single-cell flux estimation analysis
TCA: tricarboxylic acid
TEM: transmission electron microscopy
TUNEL: terminal deoxynucleotidyl transferase dUTP nick end labeling
WBC: white blood cell count
WGCNA: weighted gene co-expression network analysis
WMI: white matter injury
Zbp1: Z-DNA binding protein 1
