# Supplementary figures and images for: Neutrophil infiltration and microglial shifts in sepsis induced preterm brain injury: pathological insights
Source: Acta Neuropathol Commun. 2025 Apr 21;13:79. doi: 10.1186/s40478-025-02002-2 (PMC12010587; doi:10.1186/s40478-025-02002-2)

# CitH3

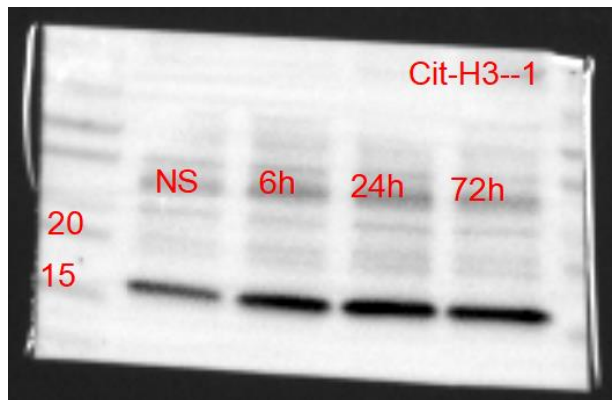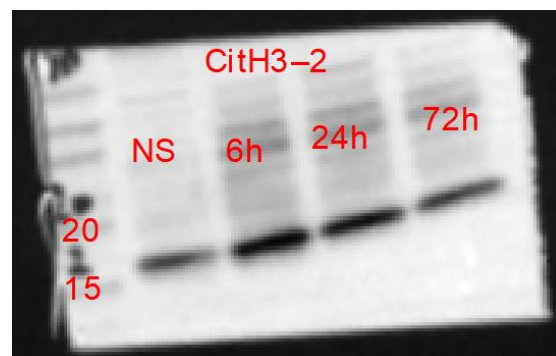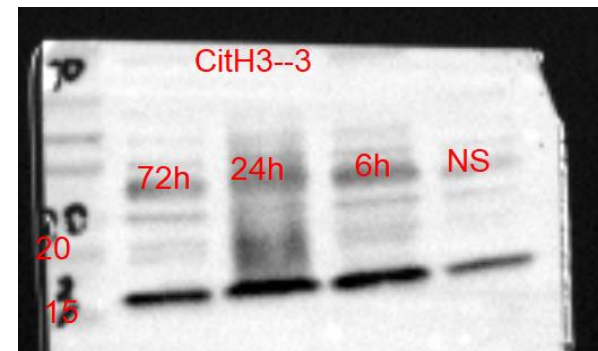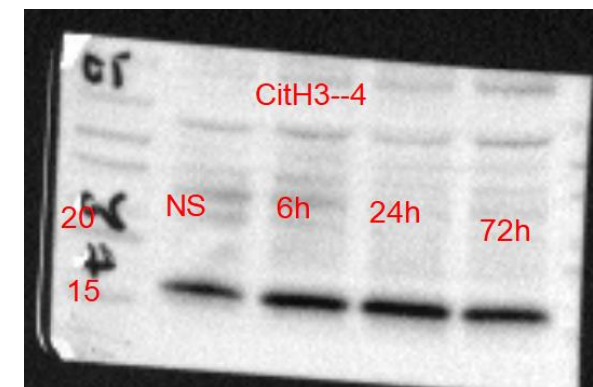

# Loading control:H3

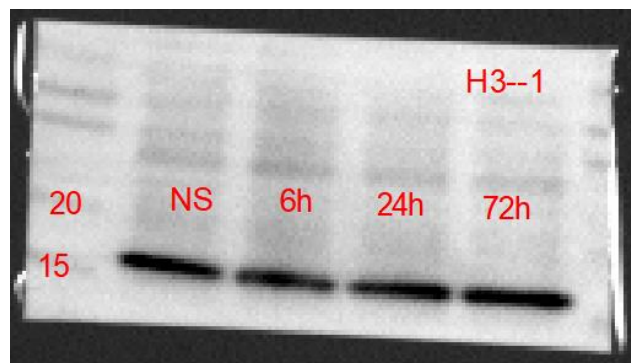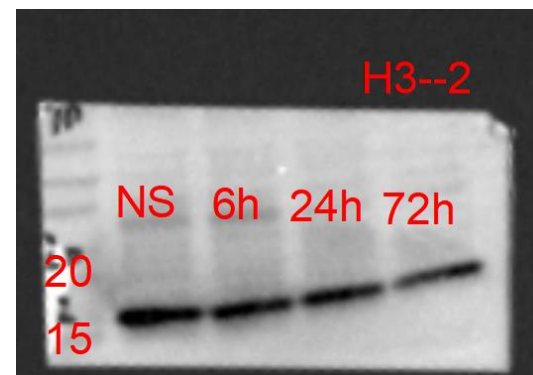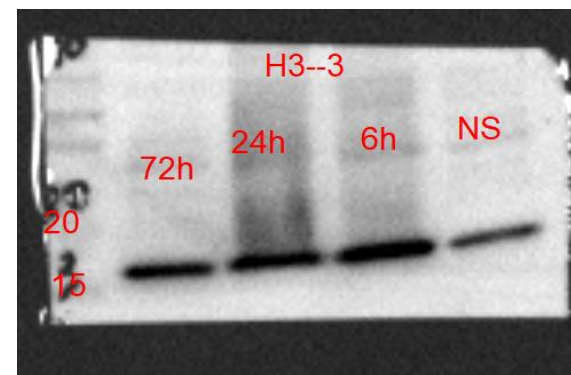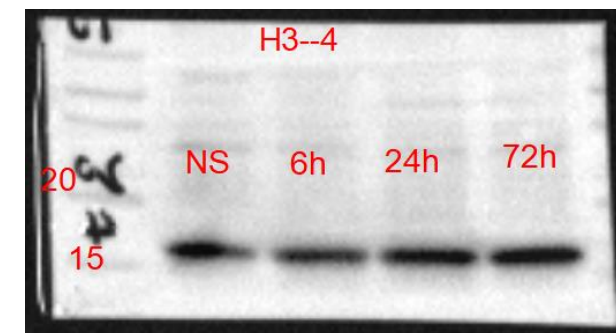

Supplement: Supplementary file 1 — Supplementary Material 1 [file 40478_2025_2002_MOESM1_ESM.pdf]
