# Supplementary material for: Neutrophil infiltration and microglial shifts in sepsis induced preterm brain injury: pathological insights
Source: Acta Neuropathol Commun. 2025 Apr 21;13:79. doi: 10.1186/s40478-025-02002-2 (PMC12010587; doi:10.1186/s40478-025-02002-2)
Supplement: Supplementary file 2 — Supplementary Material 2 [file 40478_2025_2002_MOESM2_ESM.docx]

**Supplementary materials**

**Patient characteristics**

For proteomics analysis, a total of 58 premature infants were included, with 27 subjects in the sepsis group and 29 subjects in the control group. Within the sepsis group, 8 subjects were clinically diagnosed while 19 subjects were diagnosed through laboratory testing [Gram-negative bacteria (n = 14, 73.7%), Gram-positive bacteria (n = 5, 26.3%)]. There were no statistically significant differences in the demographics between the two groups, as shown in Table S1.

**O-maze**

At P40, the anxiety levels of mice were assessed using the O-maze, an apparatus consisting of two open arms (to induce stress) and two enclosed arms (to offer protection) elevated 40 cm above the ground and arranged in a circle with a diameter of 60 cm. Animals were tested in a room with 100 lux lighting. On the test day, animals were habituated to the test room for 1 hour prior to testing. Each animal was monitored for 5 minutes via a video tracking system (Anymaze, USA) that recorded the number of entries, the duration of stay in each arm, and the distance moved in each arm. The comparison of time spent in the enclosed versus open arms served as the basis for measuring anxiety levels. Following each trial, the apparatus was cleaned with a 75% ethanol solution. The sessions were recorded on video and analyzed using Anymaze software.

**Y-maze**

At P41, short-term spatial working memory was assessed using the Y-maze. The Y-maze features three identical arms, each measuring 30 cm × 5 cm × 15 cm and set at 120-degree angles from one another. Animals were tested in a room with 100 lux lighting. On the test day, animals were habituated to the test room for 1 hour prior to testing. The Anymaze video tracking technology was used to monitor each animal, and the percentage of alternations was calculated as (number of alternations/total number of entries – 2) × 100. A higher spontaneous alternation rate indicates better spatial cognitive ability. All sessions were recorded on video and analyzed using the Anymaze software. The apparatus was cleaned with 75% ethanol after each session.

**Novel object recognition (NOR)**

At P42, recognition memory was evaluated using the NOR test. The experimental apparatus consisted of a gray opaque box measuring 45 cm × 45 cm × 45 cm. Animals were tested in a room with 100 lux lighting. On the test day, animals were habituated to the test room for 1 hour prior to testing. The NOR evaluation consisted of three parts – habituation, object familiarization, and NOR testing – with a one-hour interval between training and testing. The habituation phase consisted of placing the mice in the box and allowing them to explore the apparatus for 15 minutes, followed by the introduction of two identical blocks for object familiarization. Subsequently, during the NOR phase, one of the previously encountered blocks was substituted with a novel block, and the duration of time the mice spent exploring both the new and old blocks was recorded over a period of 10 minutes. The recognition index was calculated as (novel object time / total exploration time). The discrimination index was calculated as [(novel object time – familiar object time) / total exploration time]. This index serves as an objective measure of exploratory behavior in animals, with higher values indicating better memory and learning abilities. All sessions were recorded on video and analyzed using the Anymaze software. The apparatus was cleaned with 75% ethanol after each session.

**Social interaction test**

At P43, social interaction and novelty were assessed in a 60 cm × 40 cm × 22 cm three-chamber box. Animals were tested in a room with 100 lux lighting. On the test day, animals were habituated to the test room for 1 hour prior to testing. The experiment was performed according to the two-phase S-E protocol. The two end chambers both contained a clear Plexiglas cup. One cup contained a mouse (an age- and gender-matched mouse that had not encountered the test mice before), while the other cup was empty. In the first phase, the test mouse was placed into the central chamber, and the partitions on both sides were closed, allowing the mouse to freely move for 10 minutes to acclimate to the environment. In the second phase, the test mouse was guided into the central chamber, the partition was inserted, and a metal cage containing a stranger mouse and an empty metal cage were placed in the middle of the left and right chambers, respectively. Then, the partition was removed, allowing the subject mouse to explore freely for 10 minutes. The sociability index was calculated as (Social exploration – Empty exploration) / (Social Exploration + Empty exploration). All sessions were recorded on video and analyzed using the Anymaze software. The apparatus was cleaned with 75% ethanol after each session.

**Rotarod**

Motor function was assessed at P44 on a Rota Rod apparatus provided by (Ugo Basile, Varese, Italy). Animals were tested in a room with 100 lux lighting. On the test day, animals were habituated to the test room for 1 hour prior to testing. Before the test phase, an adaptation course was conducted at 5 revolutions per minute (rpm) for 5 minutes. Following an accelerating protocol from 4 to 40 rpm, the time taken for the mice to fall from the rods within a 300-second time limit was recorded. Each mouse underwent three trials, and the average time to fall was calculated as the individual motor behavioral threshold. The interval between each experiment is 30 minutes to allow the mice to recuperate.

**RNA sequencing sample (RNA-seq)**

For animal experiments, the isolated CD45^+^ cell precipitate from mouse brains (including the meninges and excluding the olfactory bulb and cerebellum) was homogenized in RLT lysis buffer to extract RNA for transcriptome sequencing. The count of CD45^+^ immune cells exceeded 1 × 10^5^.

**Sequencing and filtering of clean reads and sequencing depth**

A cDNA library constructed from the pooled RNA was sequenced using the Illumina NovaseqTM 6000 sequence platform. Using the Illumina paired-end RNA-seq approach, we sequenced the transcriptome, generating a total of 2 × 150 bp paired-end reads. Reads obtained from the sequencing machines included raw reads containing adapters or low quality bases that would affect the following assembly and analysis. Thus, to get high quality clean reads, reads were further filtered by Cutadapt (<https://cutadapt.readthedocs.io/en/stable/>, version: cutadapt-1.9). The parameters were as follows: 1) removing reads containing adapters; 2) removing reads containing polyA and polyG; 3) removing reads containing more than 5% of unknown nucleotides (N); and 4) removing low quality reads containing more than 20% of low quality bases (Q-value ≤20). The sequencing depth was not less than 6G**.**

**RNA-seq data analysis**

DESeq2 software was used to analyze differential gene expression between two groups. Genes with a false discovery rate (FDR) parameter below 0.05 and an absolute fold change ≥ 2 were considered to be differentially expressed. Enrichment analysis for Gene Ontology (GO) functions and Kyoto Encyclopedia of Genes and Genomes (KEGG) pathways were conducted on DEGs. Principal component analysis (PCA) was conducted with the *princomp* function in R. Volcano plots and heatmaps were generated using R software. In order to focus on annotated functional genes, the dataset was filtered using the STRING app in the Cytoscape software. For clustering and visualizing gene expression, ClusterGV was implemented (<https://github.com/junjunlab/ClusterGVis>). The ImmuCellAI databases were used to evaluate immune cell infiltration and abundance in premature brain injury.

**Immunohistochemistry and** **immunofluorescence staining**

Immunohistochemistry and immunofluorescence were conducted on P12 or P3 pups. The animals were anesthetized using sodium pentobarbital (45 mg/kg, intraperitoneally) and transcardially perfused with cold 4% paraformaldehyde. After dehydrating with graded ethanol and xylene, brain samples were embedded in paraffin and cut into 5 mm sagittal sections for the P12 sample. The P3 samples were dehydrated with a 30% sucrose solution at 4℃ until fully settled. The brains were sliced sagittally into sections of 30 mm or 5 mm using a freezing microtome (Leica Microsystems, Germany) or a semi-automatic rotary microtome (Leica Microsystems, Germany), respectively. Antigen repair was performed by immersing the sections in boiling sodium citrate buffer, and non-specific binding was blocked with 5% donkey serum in PBS. Sections were then incubated in primary antibody, washed in PBS, and incubated in secondary antibody. Antibody information can be found in Supplementary Table 3. After blocking of endogenous peroxidase activity with 3% hydrogen peroxide, chromogenic labeling was performed using 3,3'-diaminobenzidine (DAB) substrate buffer and DAB chromogen (ZSGB-BIO, ZLI-9018, China) for 5 minutes at room temperature. The slides were then scanned using a KFBIO KF-PRO-005 digital slide scanner, and images were captured with an Apotome.3 microscope (Zeiss, Germany).

TUNEL staining was performed using the TUNEL BrightGreen Apoptosis Detection Kit (Vazyme, A112) following the manufacturer’s protocol.

**Western blotting**

A total of 35 µg of denatured protein was separated on 15% SDS-polyacrylamide precast gels (YEASEN, China) and transferred to polyvinylidene difluoride membranes (Invitrogen) with a pore size of 0.22 mm. Membranes were then incubated with primary antibody, washed in TBST, and incubated with the appropriate secondary antibody. Antibody information can be found in SupplementaryTable 3. Signals were detected using the ChemiDoc XRS+ Imaging System (Bio-Rad), and the protein bands were analyzed by densitometry using the Image Lab 5.0 software.

**RT-PCR**

RNA was extracted from brain tissues 24 h post-LPS induction using TRIzol reagent (Roche, USA). The RNA concentration was determined based on the optical density (OD) of 260 nm/280 nm measured using an ultraviolet spectrophotometer. The RNA was transcribed into cDNA, and qPCR was performed using a LightCycler 96 (Roche, USA). Target gene expression was quantified using the 2-ΔΔCT method, with Gapdh mRNA as the internal control. The sample was heated to 95°C for 3 minutes, then cycled between 95°C for 10 seconds and 60°C for 30 seconds for 40 cycles. Target gene expression was quantified using the 2-ΔΔCT method, with *Gapdh* mRNA as the internal control. After the final cycle, the temperature was lowered to 65°C at a rate of 0.5°C per cycle for an additional 41 cycles, including a melting curve step.

**Supplementary Tables**

**Table S1: Baseline data of samples in the sepsis group and healthy control group**

| Characteristics | Olink sample | | | RNA-seq sample | | |
| --- | --- | --- | --- | --- | --- | --- |
|  | Sepsis group  (n = 27) | Control group  (n = 29) | *p*-value | Sepsis group  (n = 8) | Control group  (n = 8) | *p*-value |
| Gestational age (weeks) | 31.85 ± 2.60 | 32.81 ± 2.03 | 1.000 | 31.3 ± 3.96 | 32.1 ± 2.74 | 0.667 |
| Birth weight (M ± sd) | 1503.7 ± 563.1 | 1654 ± 331.6 | 0.200 | 1415 ± 430.1 | 1711 ± 393.5 | 0.173 |
| Gender [Male, n (%)] | 20(74.1) | 18 (62.1) | 0.401 | 5 (62.5) | 3 (37.5) | 0.619 |
| Singleton, n (%) | 21(77.8) | 22 (75.9) | 1.000 | 5 (62.5) | 4 (50.0) | 1.000 |
| Natural conception, n (%) | 22（81.5） | 25 (86.2) | 1.000 | 8 (100) | 8 (100) | 1.000 |
| Cesarean delivery, n (%) | 20(74.1) | 22 (75.9) | 1.000 | 5 (62.5) | 5 (62.5) | 1.000 |
| **Apgar score [M (IQR)]** | / |  |  |  |  |  |
| 1 min | 9.0(8.0,9.0) | 9.0 (7.0, 9.0) | 0.980 | 7.4 ± 2.07 | 9.1 ± 0.8 | 0.056 |
| 5 min | 9.0(9.0,10) | 10 (9.0, 10) | 0.600 | 8.6 ± 1.7 | 9.5 ± 0.5 | 0.173 |
| **Complication, n (%)** | / |  |  |  |  |  |
| NRDS | 18(66.7) | 23 (79.3) | 0.149 | 6 (75) | 6 (75) | 1.000 |
| III/IV grade IVH | 4(14.8) | 2 (6.9) | 0.666 | 0 | 0 | 1.000 |
| PVL | 2(7,4) | 1 (3.4) | 1.000 | 1 (12.5) | 0 | 1.000 |
| CHD | 21(77.8) | 23 (79.3) | 0.539 | 6 (75) | 6 (75) | 1.000 |
| BPD | 3(11.1) | 1 (3.4) | 0.604 | 3 (37.5) | 1 (12.5) | 0.569 |
| SGA | 4(14.8) | 4 (13.8) | 1.000 | 1 (12.5) | 0 | 1.000 |
| **Maternal age (years)** | 29.4 ± 4.9 | 30.5 ± 5.7 | 0.297 | 27.7 ± 6.2 | 26.3 ± 7.5 | 0.69 |
| **Antenatal glucocorticoids, n (%)** | 22(81.5) | 23 (79.3) | 0.752 | 6 (75.0) | 5 (65.2) | 1.000 |
| **Pregnancy complications** n (%) | / |  |  |  |  |  |
| Gestational hypertension | 10(37) | 9 (31) | 0.780 | 2 (25) | 3 (37.5) | 1.000 |
| GDM | 5(18.5) | 4 (13.8) | 1.000 | 0 | 0 | 1.000 |
| PROM | 4(14.8) | 7 (24.1) | 0.315 | 2 (25) | 3 (37.5) | 1.000 |
| Other | 10(37) | 12 (41.4) | 0.588 | 4 (50) | 3 (37.5) | 1.000 |
| **Outcome** | / |  |  |  |  |  |
| Survival | 26(96.3) | 29 (100) | 1.000 | 8 (100) | 8 (100) | 1.000 |
| Death | 1(3.7) | 0 | 1.000 | 0 | 0 | 1.000 |

Note: Data are expressed as the mean (M) ± standard deviation (sd) or as the percentage, and Apgar scores are expressed as interquartile range (IQR). NRDS: neonatal respiratory distress syndrome; IVH: intraventricular hemorrhage; PVL: periventricular leukomalacia; CHD: congenital heart disease; BPD: bronchopulmonary dysplasia; SGA: small for gestational age; GDM: gestational diabetes mellitus; PROM: premature rupture of membranes.

**Table S2. Primary antibodies used in the studies**

| Antibody | Species | Dilution | Conjugate/Clone | Company（Catalog#） |
| --- | --- | --- | --- | --- |
| MBP | Rabbit | 1:500 | / | Abcam (ab40390） |
| Iba-1 | Rabbit | 1:200 | / | Wako (019-19741) |
| MPO | Goat | 1:200 | / | R&D Systems (AF3667) |
| Cit-H3 | Rabbit | 1:1000 | / | Abcam (ab281584) |
| H3 | Rabbit | 1:1000 | / | Abcam (ab1791) |
| CD45 | Rat | 1:400 | Apc-cy7/30-F11 | BD Biosciences (557659) |
| CD11b | Rat | 1:400 | PE-cy7/ M1/70 | BD Biosciences (552850) |
| P2ry12 | Rat | 1:200 | APC/S16007D | BioLegend (848006) |
| Ly6g | Rat | 1:200 | BB700/1A8 | BD Biosciences (566435) |

**Table S3. Primers used in the studies**

| Gene | Forward primers | Reverse primers |
| --- | --- | --- |
| Mlkl | AATTGTACTCTGGGAAATTGCCA | AAAGACTCCTACCGTCCACAG |
| Zbp1 | CTCCTGCAATCCCTGAGAACT | GGCTACATGGCAAGACTATGTC |
| Ripk3 | TCTGTCAAGTTATGGCCTACTGG | GGAACACGACTCCGAACCC |
| Ripk2 | ATCCCGTACCACAAGCTCG | GGATGTGTAGGTGCTTCACTG |
| Slc40a1 | ACCAAGGCAAGAGATCAAACC | AGACACTGCAAAGTGCCACAT |
| Slc7a11 | GGCACCGTCATCGGATCAG | CTCCACAGGCAGACCAGAAAA |

**Table S4. Overall results of the chemokines analyses in Luminex**

| Chemokine expression in the brain | P3 | | | | P5 | | | |
| --- | --- | --- | --- | --- | --- | --- | --- | --- |
|  | M ± SD(NS) | M ± SD(LPS) | t/U value | *p*-value | M ± SD(NS) | M ± SD(LPS) | t/U value | *p*-value |
| CCL2 | 4.92 ± 1.71 | 168.2 ± 98.5 | U=94 | p=0.000 | 4.0 ± 1.1 | 7.7±3.7 | t=2.41 | p=0.03 |
| CCL3 | 24.6 ± 10.8 | 383.8 ± 332.9 | t=3.73 | p=0.003 | 26.6 ± 8.1 | 25.8 ± 16.4 | t=0.34 | p=0.73 |
| CCL4 | 2.24 ± 0.89 | 15.42 ± 17.76 | U=90 | p=0.001 | 1.75 ± 0.63 | 2.06 ± 0.74 | t=0.50 | p=0.62 |
| CCL7 | 39.8 ± 15.7 | 536.6 ± 241.5 | U=92 | p=0.001 | 29.3 ± 23.0 | 242.9 ± 194.2 | t=3.33 | p=0.006 |
| CCL11 | 6.0 ± 2.7 | 14.5 ± 5.6 | U=82 | p=0.002 | 4.95 ± 1.45 | 14.85 ± 8.85 | t=3.16 | p=0.008 |
| CCL19 | 3.62 ± 1.23 | 6.10 ± 2.06 | U=84 | p=0.005 | 2.99 ± 1.10 | 3.34 ± 1.48 | t=0.48 | p=0.63 |
| CCL20 | 2.79 ± 1.89 | 8.97 ± 4.26 | t=4.41 | p=0.000 | 3.01 ± 1.33 | 2.63 ± 1.13 | t=0.83 | p=0.41 |
| CXCL1 | 7.10 ± 3.30 | 76.4 ± 94.6 | U=92 | p=0.001 | 9.19 ± 9.05 | 8.12 ± 10.71 | U=20 | p=0.29 |
| CXCL10 | 5.08 ± 4.09 | 187.5 ± 65.9 | U=80 | p=0.003 | 3.84 ± 2.20 | 6.62 ± 6.99 | U=39 | p=0.38 |
|  | | | | | | | | |
| Chemokine expression in the blood | P3 | | | | P5 | | | |
|  | M ± SD(NS) | M ± SD(LPS) | t/U value | *p*-value | M ± SD(NS) | M ± SD(LPS) | t/U value | *p*-value |
| CCL2 | 175 ± 26 | 10530 ± 3448 | U=3.3 | p=0.000 | 197 ± 58 | 172 ± 104 | t=1.5 | p=0.14 |
| CCL3 | 77.7 ± 17.0 | 420.8 ± 167.0 | U=3.3 | p=0.000 | 87.4 ± 45.9 | 152.8 ± 108.9 | t=2.1 | p=0.04 |
| CCL4 | 59.5 ± 14.8 | 354.5 ± 149.3 | U=3.2 | p=0.000 | 56.4 ± 37.4 | 105.5 ± 65.0 | t=0.6 | p=0.53 |
| CCL7 | 7651 ± 1205 | 25431 ± 8462 | U=2.6 | p=0.006 | 95 ± 123 | 381 ± 534 | t=1.9 | p=0.06 |
| CCL11 | 3738 ± 1062 | 1631 ± 1012 | t=3.9 | p=0.002 | 2263 ± 2060 | 5122 ± 3481 | U=1.0 | p=0.36 |
| CCL19 | 102.2 ± 13.5 | 294.6 ± 87.8 | t=5.7 | p=0.000 | 43.3 ± 22.5 | 81.3 ± 44.2 | t=1.7 | p=0.10 |
| CCL20 | 127.2 ± 34.0 | 1093 ± 1116 | t=2.4 | p=0.028 | 88.0 ± 59.3 | 105.3 ± 64.9 | t=0.5 | p=0.58 |
| CXCL1 | 239 ± 112 | 3729 ± 2327 | U=3.1 | p=0.001 | 41.6 ± 25.9 | 51.1 ± 33.7 | t=0.4 | p=0.64 |
| CXCL10 | 185 ± 23 | 3729.5 ± 1571 | U=3.0 | p=0.001 | 1658 ± 1424 | 780 ± 687 | t=1.3 | p=0.19 |

**Table S5. Overall results of the chemokines analyses in Olink**

|  | HC *vs* Sepsis | | | | |
| --- | --- | --- | --- | --- | --- |
|  | M ± SD(HC) | M ±SD(Sepsis) | t/U value | *p*-value | |
| CCL2 | 12.01±0.47 | 12.21±1.06 | U=1.1 | | p=0.261 |
| CCL3 | 5.91±0.56 | 6.76±1.11 | U=2.9 | | p=0.003 |
| CCL4 | 6.78±0.65 | 7.06±0.86 | U=1.2 | | p=0.19 |
| CCL7 | 1.26±0.43 | 2.30±1.31 | t=3.2 | | p=0.001 |
| CCL11 | 6.93±0.49 | 6.54±0.57 | t=2.7 | | p=0.009 |
| CCL19 | 8.67±0.65 | 8.67±0.87 | t=0.9 | | p=0.99 |
| CCL20 | 8.70±0.86 | 9.41±1.48 | t=1.4 | | p=0.14 |
| CXCL1 | 9.83±0.93 | 10.03±0.79 | t=0.8 | | p=0.379 |
| CXCL10 | 9.07±1.05 | 10.19±1.63 | U=2.9 | | p=0.003 |

**Supplementary Figures
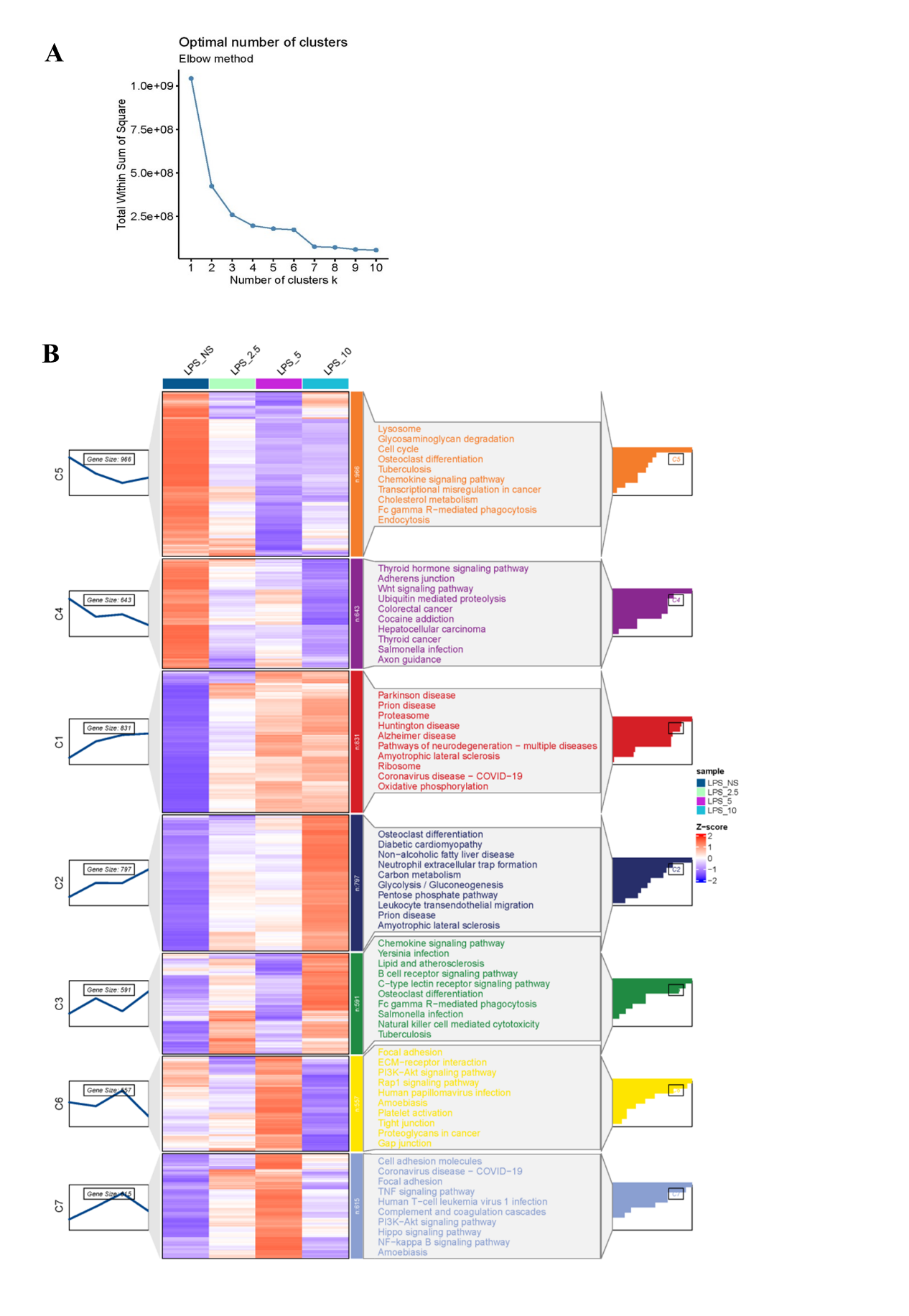
**

**Fig. S1. Clusters were classified through Mfuzz clustering to depict the dynamic expression or co-regulation patterns in the four groups**. **A.** Mfuzz clustering analysis identified seven distinct clusters. **B**. The heatmap shows the top 10 KEGG pathway names and their associated z-scores (blue for negative z-scores, red for positive z-scores).

**
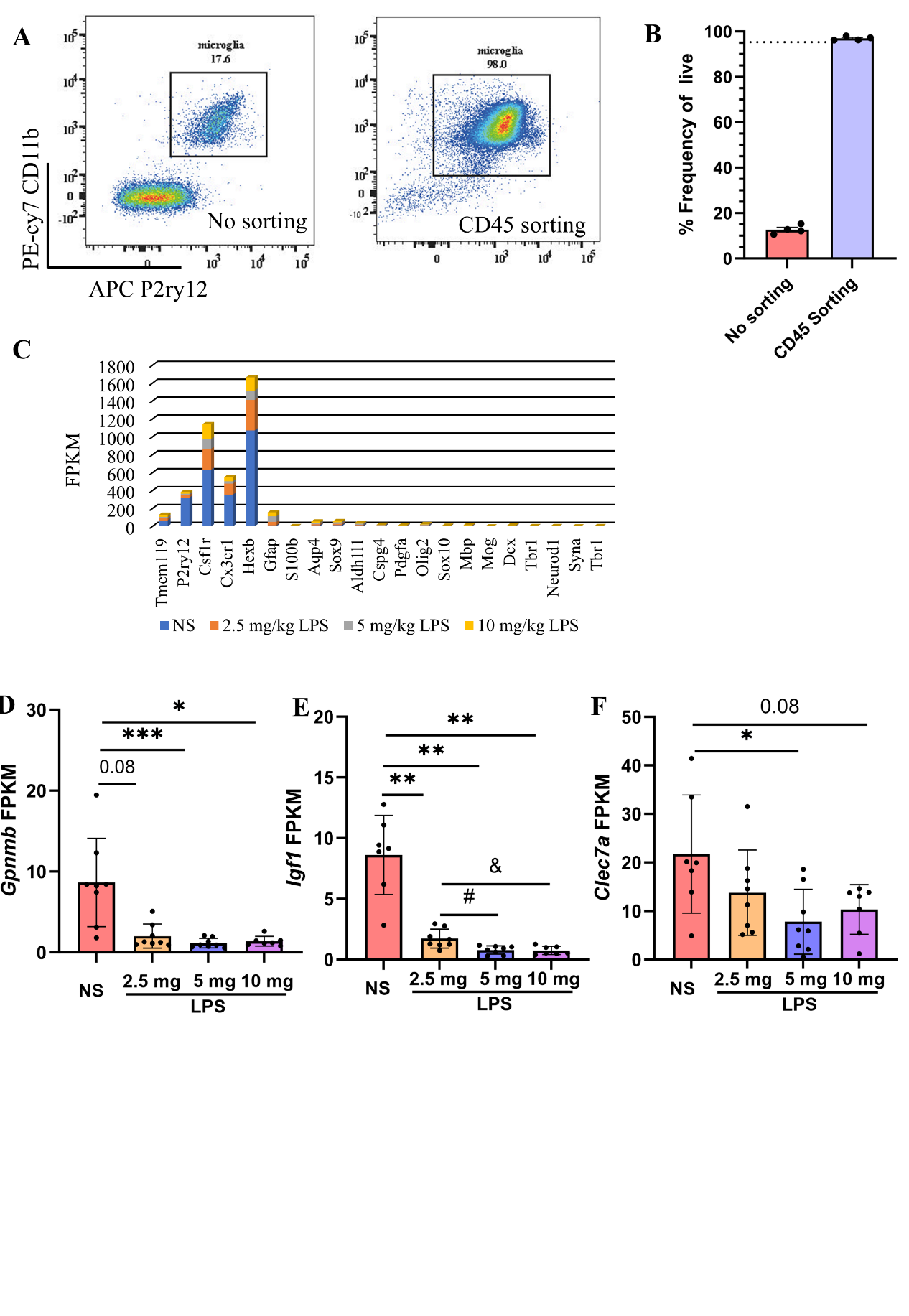
Fig. S2. CD45 sorting and gene expression profiles in response to LPS stimulation at different doses**. **A**. The gating strategy for analyzing immune cells from the periphery. **B**. The frequency of CD45^+^cells (Live/CD411b^+^/P2ry12^+^) was analyzed by flow cytometry. n = 4; **C**. The superimposed histogram showing the gene FPKM values of neuron and glial cell marker genes (n = 8/group) at different doses of LPS. **D-F**. Differences in the expression of selected CD11c^+^ microglia genes between the NS and LPS groups. (Kruskal–Wallis test with Dunn post hoc test and Welch one-way ANOVA test with Games–Howell: *Gpnmb*: H = 16.21, *P* = 0.001; *Igf1*: F3,26 = 6.7, *P* = 0.001; *Clec7a*: F3,26 = 3.6, *P* = 0.02; n = 7–8/group, ***P* < 0.01, ^#^*P* < 0.05, 2.5 mg/kg LPS vs. 5 mg/kg LPS, ^&^*P* < 0.05, 2.5 mg/kg LPS vs. 10 mg/kg LPS).
